# Presynaptic Mu Opioid Receptors Suppress the Functional Connectivity of Ventral Tegmental Area Dopaminergic Neurons with Aversion-Related Brain Regions

**DOI:** 10.1523/JNEUROSCI.1194-24.2025

**Published:** 2025-06-13

**Authors:** Yichen Wu, Tamara Perez-Rosello, Rajeshwar Awatramani, Dalton James Surmeier

**Affiliations:** ^1^Departments of Neuroscience, Northwestern University, Chicago, Illinois 60611; ^2^Neurology, Feinberg School of Medicine, Northwestern University, Chicago, Illinois 60611

**Keywords:** aversion, dopaminergic neuron, negative reinforcement, opioids, ventral tegmental area

## Abstract

Opioid abuse poses a major healthcare challenge. To meet this challenge, the brain mechanisms underlying opioid abuse need to be more systematically characterized. It is commonly thought that the addictive potential of opioids stems from their ability to enhance the activity of ventral tegmental area (VTA) dopaminergic neurons. Indeed, activation of mu opioid receptors (MORs) disinhibits VTA dopaminergic neurons projecting to the nucleus accumbens, providing a substrate for the rewarding effects of opioids. However, the abuse potential of opioids has also been linked to their ability to suppress pain and aversive states. Although medial VTA dopaminergic neurons are commonly excited by aversive stimuli, the effects of MOR signaling on this circuitry have not been systematically explored. To fill this gap, a combination of anatomical, optogenetic, and electrophysiological approaches were used to study the afferent circuitry of paranigral VTA (pnVTA) dopaminergic neurons and its modulation by MOR signaling in male and female mice. These studies revealed that aversion-linked glutamatergic neurons in the lateral hypothalamus, ventrolateral periaqueductal gray, and lateral habenula innervated a subset of pnVTA dopaminergic neurons and that activation of presynaptic MORs suppressed their ability to drive pnVTA spiking. A distinct set of pnVTA dopaminergic neurons were innervated by lateral hypothalamus GABAergic neurons, which also were subject to MOR modulation. Thus, MORs robustly inhibit the ability of brain circuits coding aversive states to drive the activity of pnVTA dopaminergic neurons, suggesting that the addictive potential of opioids may stem in part from their ability to act as negative reinforcers.

## Significance Statement

Opioid abuse is a severe, worldwide problem. The ventral tegmental area (VTA) is part of the brain circuitry underlying opioid dependence. Previous work has shown that opioid activation of mu opioid receptors (MORs) suppresses GABAergic inhibition of VTA dopaminergic neurons, enhancing dopamine release and reward. However, the central mechanisms responsible for the ability of opioids to alleviate pain are less clear. Here we demonstrate that MORs suppress the ability of neurons in three aversion-related brain regions to drive spiking in dopaminergic neurons located in the paranigral region of the VTA—a subregion linked to pain perception. Thus, these studies add a new dimension to our understanding of the central actions of opioids and their potential role in opioid abuse.

## Introduction

Opioid abuse is a severe, worldwide problem, with over 26 million deaths/year attributed to overdosing (Strang et al., 2020; Monroe and Radke, 2023). The current opioid abuse “epidemic” stems in part from their use in pain management (Koob, 2021; Pantazis et al., 2021). Long-term use of opioids can lead to dependence and addiction (Ballantyne and LaForge, 2007; Juurlink and Dhalla, 2012; Volkow et al., 2018).

The ventral tegmental area (VTA) is part of the brain circuitry underlying opioid dependence and other forms of addiction (Fields and Margolis, 2015; Pascoli et al., 2015). VTA dopaminergic neurons play a key role in hedonia, reward learning, and avoidance, which are central to the processes underlying drug addiction (Juarez and Han, 2016; Stuber, 2023). Work over the last decade has established that mu opioid receptors (MORs) suppress GABAergic inhibition of VTA dopaminergic neurons (and attenuate GABAergic inhibition of glutamatergic inputs; Chen et al., 2015), enhancing dopamine (DA) release in forebrain circuits (Matsui and Williams, 2011; Li et al., 2019; Taylor et al., 2019; Jhou, 2021). DA release in regions like the nucleus accumbens (NAc) is firmly linked to reward, providing a compelling mechanism underlying the addictive potential of opioids (Zocchi et al., 2003; Nieh et al., 2016; Corre et al., 2018).

However, there are several reasons to think that the circuitry underlying opioid addiction, particularly within the context of pain, is more complicated. Lineage tracing, molecular profiling, and connectomic mapping have demonstrated that VTA dopaminergic neurons are heterogeneous (Lammel et al., 2014; Morales and Margolis, 2017; Poulin et al., 2018; de Jong et al., 2022). Dopaminergic neurons subject to MOR-mediated disinhibition reside largely in the lateral regions of the VTA (Ambroggi et al., 2008, 2011; Nicola, 2010; Nieh et al., 2016), although a subset of these also are found in the medial VTA (Corre et al., 2018). These neurons are typically excited by rewarding, appetitive stimuli and inhibited by aversive events (Eshel et al., 2016; de Jong et al., 2019; Yuan et al., 2019). In contrast, dopaminergic neurons in the medial, paranigral region of the VTA (pnVTA) are commonly excited by aversive stimuli and project to the ventromedial shell of the NAc (vmNAc), rather than the core of the NAc (cNAc; Brischoux et al., 2009; Moriya et al., 2018; de Jong et al., 2019). The origin of the aversive input to pnVTA dopaminergic neurons is poorly defined. One potential source arises from glutamatergic neurons in the lateral hypothalamus (LH). Stimulation of LH glutamatergic neurons innervating the medial VTA is aversive and triggers avoidance (de Jong et al., 2019). In contrast, stimulating LH GABAergic neurons innervating the lateral VTA is rewarding and promotes place preference by inhibiting local GABAergic interneurons (Nieh et al., 2016). Other brain regions involved in signaling aversive events, like the ventrolateral periaqueductal gray (vlPAG) and lateral habenula (LHb), have been implicated in the regulation of the lateral VTA (Matsumoto and Hikosaka, 2007; Baker et al., 2016; St Laurent et al., 2020), but their roles in controlling the pnVTA have not been systematically explored. Furthermore, the impact of MOR signaling pathways on the regulation of aversive signaling in the pnVTA has not been rigorously characterized.

Our experiments revealed that glutamatergic neurons in the LH, vlPAG, and LHb robustly innervate overlapping populations of pnVTA dopaminergic neurons and are capable of rapidly accelerating spiking rate. Moreover, each of these afferents is invested with presynaptic MORs, which produce a long-lasting inhibition of transmitter release. In contrast, LH GABAergic neurons innervate a distinct population of pnVTA dopaminergic neurons. But, like glutamatergic terminals, LH GABAergic terminals are potently suppressed by MOR stimulation. Taken together, these results suggest that the abuse potential of opioids stems not just from MOR-mediated disinhibition of lateral VTA dopaminergic neurons coding reward, but also from MOR-mediated suppression of aversive signaling to medial VTA dopaminergic neurons and negative reinforcement.

## Materials and Methods

### Animals

All electrophysiology studies were conducted in male DAT-Cre × Ai14-tdTomato-flox transgenic mice (11–14 weeks). The effect of opioids on presynaptic neurotransmission was examined in both male and female DAT-Cre × Ai14-tdTomato-flox transgenic mice. DAT-Cre × Ai14-tdTomato-flox transgenic mice were made in our laboratory by backcrossing B6.SJL-Slc6a3^tm1.1(cre)Bkmn^/J mice (common name = DAT^IRES*cre*^) purchased from the Jackson Lab (strain ID: 006660) and B6;129S6-Gt(ROSA)26Sor^tm14(CAG-tdTomato)Hze^/J mice (common name = Ai14; the Jackson Lab, strain ID: 007908), which were used for identification of dopaminergic neurons in the VTA. Animals were group-housed with littermates, with food and water available *ad libitum* and with a 12** **h light/dark cycle (7 A.M.–7 P.M.). Adult (7–8 weeks) male Ai14 transgenic mice (the Jackson Lab, strain ID: 007908) were used for viral retrograde mapping. All studies were performed following the guidelines of the Animal Care and Use Committee of Northwestern University and the National Institutes of Health on animal care.

### Stereotaxic surgeries

All surgeries for stereotaxic injections of recombinant adeno-associated virus (AAV) carrying channelrhodopsin2 (ChR2), ChrimsonR, or Cre recombinase were conducted in adult mice (7–9 weeks). Mice were anesthetized with isoflurane (2%) and were subcutaneously injected with bupivacaine solution (0.15** **mg/ml) into the incision area. A small hole was drilled through the skull for virus injection. Mice were placed on a heating pad throughout the surgery to avoid hypothermia. For AAV9-hSyn-hChR2(H134R)-EYFP (Addgene, #26973-AAV9) injections, 100** **nl of virus solution (titer: 2.1 × 10^12^** **GC/ml) was injected into the LHb (bregma:−1.70** **mm, lateral: −0.37** **mm, ventral: −2.60** **mm), LH (bregma:−1.06** **mm, lateral: −1.08** **mm, ventral: −5.2** **mm), or vlPAG (bregma: −4.84** **mm, lateral: −0.70** **mm, ventral: −2.64** **mm) of DAT-Cre × Ai14-tdTomato-flox transgenic mice using a IM 300 microinjector (NARISHIGE) at 100** **nl/min. The injection needle was withdrawn 5–10** **min after the end of the infusion. The incision was then closed with a suture. Mice were kept on the heating pad until they recovered from anesthesia. For two-color optogenetics, 100** **nl of AAV9-Syn-ChrimsonR-tdT virus (Addgene, #59171-AAV9; titer: 2.6 × 10^13^** **GC/ml) and AAV9-hSyn-hChR2(H134R)-EYFP virus (titer: 2.1 × 10^12^** **GC/ml) were injected into the LH and vlPAG of DAT-Cre × Ai14-tdTomato-flox transgenic mice, respectively. Electrophysiology experiments were performed 3–4 weeks after stereotactic injection. Injection sites were confirmed in all animals by preparing coronal sections (50–200** **mm) and performing confocal imaging. For retrograde mapping, 50** **nl of AAVrg-hSyn-Cre-WPRE-hGH virus (Addgene, #105553-AAVrg; titer: 1.8 × 10^12^** **GC/ml) was injected into the pnVTA (bregma: −3.40** **mm, lateral: −0.25** **mm, ventral: −4.47** **mm) of Ai14 transgenic mice. Confocal imaging was performed 3 weeks later.

### Electrophysiology

#### Brain slices preparation

Coronal brain slices containing the pnVTA (200** **µm) were obtained from virus-injected transgenic mice. Mice were anesthetized with ketamine/xylazine and perfused transcardially with ice-cold artificial cerebrospinal fluid (aCSF), containing the following (in mM): 125 NaCl, 2.5 KCl, 1.25 NaH_2_PO_4_, 2.0 CaCl_2_, 1.0 MgCl_2_, 25 NaHCO_3_, and 25 glucose, saturated with 95% O_2_ and 5% CO_2_. Mice were rapidly decapitated, and brains were extracted and sliced in the oxygenated, ice-cold, cutting solution containing the following (in mM): 210 sucrose, 2.5 KCl, 1.25 NaH_2_PO_4_, 0.5 CaCl_2_, 25 NaHCO_3_, 10 MgSO_4_, 0.4 ascorbic acid, and 10 glucose, by using a VT1200S Vibratome (Leica Microsystems). Slices were transferred to a holding chamber and incubated in the aCSF at 34°C for 30 min and then stored at room temperature for another 30 min before electrophysiological recordings. The extracellular aCSF was saturated with 95 O_2_/5% CO_2_ to maintain oxygenation and a pH of 7.4.

#### Visualized whole-cell patch-clamp recording ex vivo

Slices were visualized using an upright microscope (Olympus) equipped with infrared differential interference contrast optics and with a USB 3.0 digital camera (THORLABS). Cell-attached and whole-cell patch-clamp recordings were made on tdTomato-expressed dopaminergic neurons in the pnVTA of DAT-Cre × Ai14-tdTomato-flox transgenic mice. For excitatory postsynaptic currents (EPSCs) recording, GABA_A_ and GABA_B_ antagonists (10 μM SR 95531, 1 μM CGP55845) were bath applied. For IPSC recording, DNQX (10 μM) was bath applied to block AMPA receptors. A combination of TTX (1 μM) and 4-AP (10 μM) was used to examine monosynaptic connections between the LH and pnVTA. DAMGO (1 μM) and naloxone (10 μM) were used to activate and inhibit μ-opioid receptors, respectively. Recordings were made at 32–34°C using a MultiClamp 700B amplifier with pClamp 10.7 software (Molecular Devices). Signals were filtered at 1–2 kHz and digitized at 10 kHz with a Digidata 1550B (Molecular Devices). Patch electrodes were pulled on a Flaming–Brown horizontal puller (P-97; Sutter Instrument) from filamented, thick-wall borosilicate glass (1.5** **mm O.D.; Sutter Instrument). For cell-attached recordings, pipette resistance was typically 3–5** **MΩ when filled with an internal solution consisting of the following (in mM): 135 KMeSO_4_, 5 KCl, 5 HEPES, 2 ATP-Mg, 0.5 GTP-Na, 10 sodium phosphocreatine, and 0.05 EGTA; pH 7.30; 300** **mOsm. For whole-cell recordings, pipette resistance was typically 2–3** **MΩ when filled with an internal solution consisting of the following (in mM): 120 CsMeSO_3_, 5 NaCl, 10 TEA-Cl, 10 HEPES, 3 QX-314, 4 ATP-Mg, 0.3 GTP-Na, and 0.2 EGTA, pH 7.30; 300 mOsm. The liquid junction potential in our recording ACSF using this internal solution was 9** **mV and not corrected.

#### Optically evoked synaptic responses

Cell-attached recordings were performed to determine whether inputs were excitatory or inhibitory. Inputs from LH, vlPAG, or LHb were determined by a ChR2 stimulation using 473** **nm wavelength light pulses (2** **s train stimulation at 20** **Hz with 5** **ms pulse duration, holding at −70** **mV) after a 10** **s recording for spontaneous spiking. Excitatory inputs increased the spiking rate during light stimulations while inhibitory inputs caused a spiking pulse. After an initial characterization in cell-attached mode, neurons were frequently transitioned to whole-cell mode to record synaptic currents and characterize their properties. Postsynaptic currents were evoked in response to 0.05** **Hz ChR2 stimulation (0.3–1** **ms pulse duration, holding at −70** **mV). For example, optogenetic activation of LH axons either excited or inhibited pnVTA dopaminergic neurons in the cell-attached mode. Once the response type was determined, the cell was transitioned to whole-cell mode and the ability of bath-applied ionotropic glutamate or GABA receptor antagonists to inhibit synaptic currents was determined (based upon the response in cell-attached mode). For paired-pulse recording, two identical light pulses (0.3–1** **ms) with a 50** **ms interval were evoked by the ChR2 stimulation. The paired-pulse ratio (PPR) of the second postsynaptic current (PSC) over the first PSC was calculated to quantify the probability of presynaptic vesicle release. The access resistance was monitored by a hyperpolarizing step of −10** **mV with each sweep. The data were discarded if the access resistance changed by >20% during the recordings. For two-color recordings, ChrimsonR was excited using a 615/660** **nm LED, and ChR2 was excited by a 430/477** **nm LED by the X-Cite Turbo six-LED unit system (Excelitas Technologies). After establishing stable whole-cell recordings, the two opsins were sequentially stimulated (2** **s optical stimulation at 20** **Hz with a 1** **ms pulse duration) in the same pnVTA dopaminergic neurons, with a 1** **min interval between stimulations. The convergence of inputs was assessed. With this system, the timing, intensity, and duration of each pulse can be independently varied to minimize excitation of ChrimsonR while activating ChR2.

### Aversive stimulation

Fifty nanoliters of AAVrg-hSyn-Cre-WPRE-hGH virus (Addgene, #105553-AAVrg; titer: 1.8 × 10^12^** **GC/ml) were injected into the pnVTA of Ai14 transgenic mice 3 weeks before the experiment. Bedding and hideout were placed in the procedure cage. Before the experiment, mice were placed in the procedure cage for a 30** **min acclimatization.

#### Tail pinch

Mice were given 10 tail pinches using forceps (1** **s duration, 60** **s intervals between pinches). For the control group, mice were allowed to move freely in the cage for 10 min (H. Yang et al., 2021).

#### Predator odor 2,5-dihydro-2,3,5-trimethylthiazoline

A 50** **ml centrifuge tube containing filter paper was placed on the bedding. The cage was placed in the fume hood to prevent the dissipation of 2,5-dihydro-2,3,5-trimethylthiazoline (TMT) odor. Water (control) or TMT solution (10** **μl) was added to the filter paper, and the mice were allowed to be exposed for 15 min (Ornelas and Besheer, 2024).

#### Formaldehyde

The cage was placed in the fume hood to prevent the dissipation of formaldehyde (FA). A cotton swab dipped in water (control) or 6% FA solution was placed on the bedding, and the mice were allowed to be exposed for 15 min (de Jong et al., 2019).

Mice were perfused with 4% paraformaldehyde (PFA, Electron Microscopy Sciences) 45 min later for immunohistochemistry.

### Immunohistochemistry and confocal imaging

To visualize EYFP+ axons and postsynaptic pnVTA dopaminergic neurons, brain slices were fixed with 4% PFA after electrophysiological recording. For retrograde labeling and aversive stimulation, anesthetized Ai14 mice were perfused with phosphate-buffered saline (PBS, Sigma-Aldrich) followed by 4% PFA. Brains were extracted, put in PFA overnight, and washed and stored in PBS. Coronal brain slices (50** **µm) containing areas of interest were obtained using a VT1200S Vibratome. Slices were incubated overnight in primary antibody [c-Fos (9F6) Rabbit mAb; 1:1,000, Cell Signaling Technology). On the second day, slices were stained in secondary antibody [Alexa Fluor 488 goat anti-rabbit IgG (H + L); 1:500, Thermo Fisher Scientific] for 2 h. Slices were incubated in 5** **µg/ml DAPI for 5 min followed by a 15 min wash with PBS. Image acquisition was performed with an Olympus FV10i confocal laser scanning microscope. To characterize the aversive inputs to pnVTA, confocal imaging for retrograde labeling and aversive stimulation was acquired with the same laser settings, pinhole, and gain. For the tail pinch, TMT, and FA experiments, recordings were obtained from three control mice and three experimental mice. Each imaging site included 20 1 μm sections of regions of interest.

### Pharmacological reagents and chemicals

Reagents were purchased from Sigma except for QX-314 (Tocris#2313), GABAzine (SR95531, Tocris#1262), CGP55845 (Tocris#1248), DNQX (Tocris#2312), DAMGO (Tocris#1171), naloxone (Tocris#0599), and 4-aminopyridine (4-AP, Tocris#0940). TMT (≥90% purity, 1 g) was purchased from BioSRQ. c-Fos (9F6) Rabbit mAb (cFos; #2250S) was purchased from Cell Signaling Technology. Alexa Fluor 488 goat anti-rabbit IgG (H + L) was purchased from Thermo Fisher Scientific.

### Experimental design and statistical analysis

Data analyses were conducted with ImageJ, Clampfit 10.7 (Molecular Devices), Igor Pro 9.00 (WaveMetrics), and GraphPad Prism 9.0 (GraphPad Software). Bar plots (mean ± SEM) were used for EPSC or IPSC blockade experiments. Box plots were used for all the other data: the thick line as the median, the box edges showing first/third quartiles, and the whiskers representing minimum/maximum values. Statistical analysis was performed with GraphPad Prism 9.0. Nonparametric Wilcoxon matched-pairs signed-rank test was used for the comparison of two paired groups. Mann–Whitney test was used for the comparison of two unpaired groups. RM one-way ANOVA with Tukey's correction was used for multiple comparisons of paired data. The Kruskal–Wallis test with multiple comparisons was used for multiple comparisons of unpaired data. Statistical tests were indicated in the main text. Neurons or brain slices for electrophysiology are collected from at least five mice, and one or two brain slices per mouse. Imaging sites for cFos staining are collected from six mice (three control and three stimulated mice). The probability threshold for statistical significance was *p* < 0.05 (*p < 0.05, **p < 0.01).

## Results

### LH glutamatergic and GABAergic neurons innervate different pnVTA dopaminergic neurons

**Figure 1.**
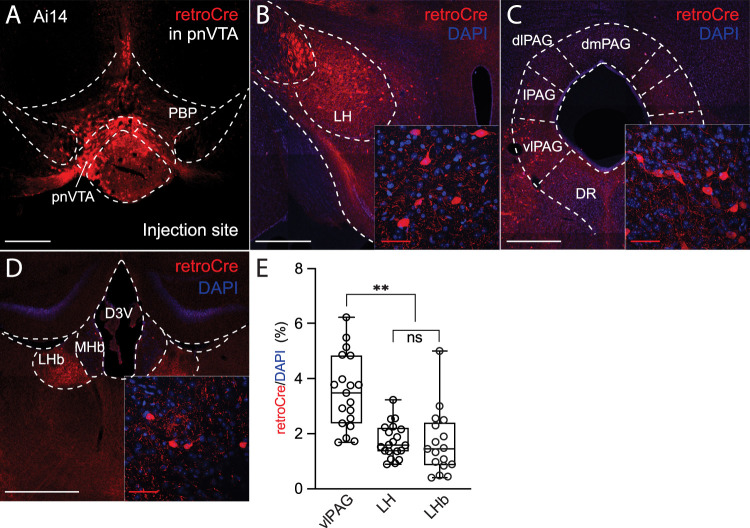
LH, vlPAG, and LHb neurons innervate pnVTA. ***A***, After the injection of retrograde AAVrg-Cre into the pnVTA of the Ai14 mice (*n* = 4), tdTomato signal was restricted to the pnVTA (injection site; arrow head). ***B*–*D***, the retrograde labeling and the DAPI staining were detected in the LH (Fig. 1*B*), vlPAG (Fig. 1*C*), and LHb (Fig. 1*D*). An enlarged view of the region of interest is presented as an inset. ***E***, Data summary of the retrogradely labeled neuron ratio (LH, *n* = 20 imaging sites; vlPAG, *n* = 19 imaging sites; LHb, *n* = 17 imaging sites). pnVTA, paranigral nucleus of the ventral tegmental area; LH, lateral hypothalamus; vlPAG, ventrolateral periaqueductal gray; LHb, lateral habenula. Scale bar, 500** **µm (white); 50** **µm (red; inset).

To determine its afferent innervation, a retrograde adeno-associated viral (AAV) vector carrying cre recombinase (cre) expression construct was injected into the pnVTA of Ai14 reporter mice; in this reporter line, cre drives the expression of tdTomato. Three weeks after AAV injection, mice were killed, and serial section confocal microscopy was performed to determine those neuronal populations projecting to pnVTA (Fig. 1*A*, images of injection site). Several brain regions were robustly labeled using this approach, including the LH, LHb, and the vlPAG (Fig. 1*B*–*D*). To estimate the proportion of neurons in these regions projecting to the pnVTA, retrograde labeling was combined with DAPI staining. The percentage of retrograde-labeled neurons (retroCre/DAPI) in the vlPAG (Fig. 1*E*; 3.5 ± 0.3%; *n* = 19 imaging sites) was higher than that in the LH (1.7 ± 0.1%; *n* = 20 imaging sites; Kruskal–Wallis test with multiple comparisons, *p* = 0.0002) and LHb (1.7 ± 0.3%, *n* = 17 imaging sites; *p* < 0.0001).

**Figure 2.**
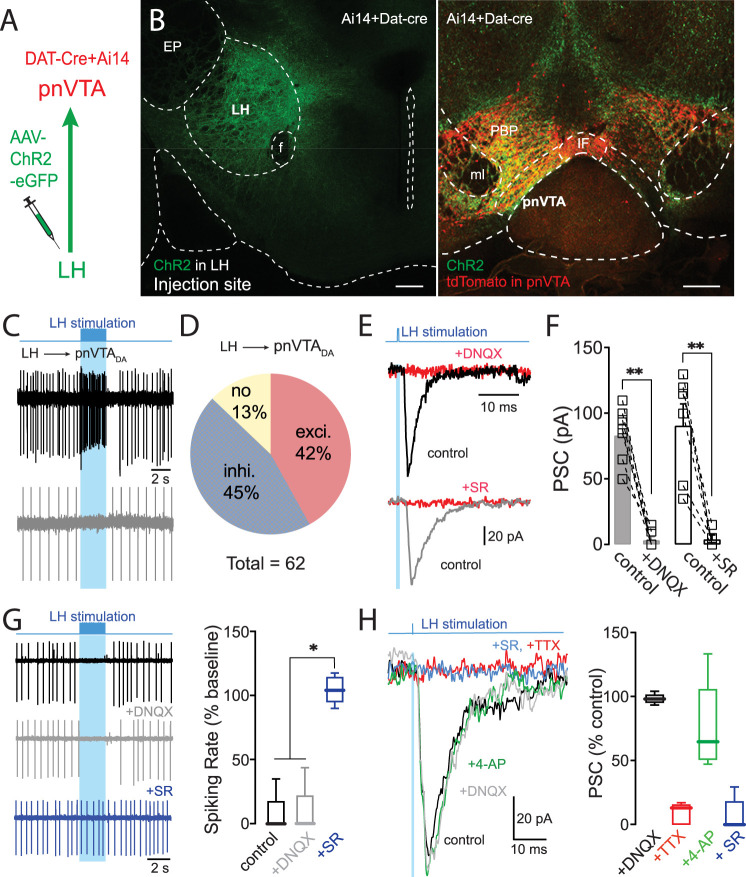
Optogenetic activation of LH axons evoked EPSCs and IPSCs in pnVTA dopaminergic neurons. ***A***, Diagram of viral injection and patch recording. ***B***, After the injection of AAV9-ChR2-EGFP into the LH of the Ai14 X DAT-cre mice, EGFP signal was detected in the LH (injection site, left), and tdTomato and EGFP labeling was detected in the pnVTA (right). Scale bar, 200** **µm. ***C***, Example of burst firing or spiking pause evoked in response to a 2** **s optical stimulation of LH axons (20** **Hz with 5** **ms pulse duration) in pnVTA dopaminergic neurons (*n* = 62 cells; black, excited; gray, inhibited). ***D***, A pie chart illustrating the ratio of neuronal responses to LH stimulation. 87% of pnVTA dopaminergic neurons responded to LH stimulation (42%, excited; 45%, inhibited). ***E***, Example of EPSCs (black) and IPSCs (gray) evoked in response to 0.05** **Hz optical stimulation (0.3–1** **ms pulse duration) of LH axons. DNQX (10** **µM) and SR (10** **µM) blocked the EPSCs and IPSCs, respectively. ***F***, Data summary (EPSC, *n* = 7 cells; IPSC, *n* = 6 cells). ***G***, The LH-induced pause in spiking remained in the presence of DNQX (10** **µM, gray), but the pause was blocked by the GABAaR antagonist SR (10** **µM, blue). Data summary (*n* = 5 cells, right). ***H***, Example of optically induced IPSC in pnVTA dopaminergic neuron (left). The IPSC was resistant to DNQX (10** **µM) and remained in the presence of TTX (1** **µM, red) and 4-AP (10** **µM, green). IPSC was blocked by SR (10** **µM, blue). Data summary (*n* = 7 cells, right). The characteristics of LH inputs is shown in the Extended Data Figure 2-1.

To assess the functional connectivity of LH neurons with pnVTA dopaminergic neurons, a combination of optogenetic and electrophysiological approaches were used. The LH of DAT-Cre × Ai14 mice was injected with an AAV carrying a channelrhodopsin2 (ChR2) expression construct (Fig. 2*A*,*B*). Three weeks later, mice were killed and brain slices were prepared. Cell-attached recordings were made from visually identified, tdTomato-expressing dopaminergic neurons in the pnVTA. These neurons were spontaneously active. Roughly 42% of this sample (26/62) were excited by optogenetic stimulation of LH axons, whereas ∼45% (28/62) were inhibited (Fig. 2*C*,*D*). Both LH inputs were robust: in pnVTA dopaminergic neurons that were excited by LH burst stimulation, the rate increased by 730 ± 147% (*n* = 26 cells); in those neurons that were inhibited by burst stimulation, the rate dropped by ∼80% (*n* = 27 cells; Extended Data Fig. 2-1*A*).

10.1523/JNEUROSCI.1194-24.2025.f2-1 Figure 2-1 The characteristics of LH inputs. A, Quantification of the spiking rate induced by LH stimulation. Excited (n = 26 cells) and inhibited (n = 28 cells) spiking in response to 2  s optical stimulation (40 pulses at 20  Hz; 1  ms duration) of LH axons. Each spiking rate was normalized to the baseline spiking rate. B, The 10-90% rise time of the EPSCs (n = 6 cells) and IPSCs (n = 5 cells). C, Coefficient of variation (CV) - mean plot of the pace-making interval (black, excited, n = 20 cells; gray, inhibited, n = 21 cells). Download Figure 2-1, TIF file.

To better characterize the LH synapses, whole-cell voltage-clamp recordings with a Cs^+^-containing internal solution were made from pnVTA dopaminergic neurons after determining their response to LH stimulation in cell-attached mode. Excitatory postsynaptic currents (EPSCs) were blocked by 6,7-dinitroquinoxaline-2,3-dione (DNQX, 10** **µM; Fig. 2*E*,*F*; control: 84 ± 9** **pA; +DNQX: 4 ± 3** **pA; *n* = 7 cells; Wilcoxon matched-pairs test, *p* = 0.0156). Inhibitory postsynaptic currents (IPSCs) were blocked by SR 95531 (SR; 10** **µM; Fig. 2*E*,*F*; control: 91 ± 17** **pA; +SR: 4 ± 3** **pA; *n* = 6 cells; Wilcoxon matched-pairs test, *p* = 0.0312). The rise times of EPSCs and IPSCs were similar (Extended Data Fig. 2-1*B*; EPSC: 1.14 ± 0.12** **ms, *n* = 6 cells; IPSC: 1.30 ± 0.21** **ms, *n* = 5 cells; Mann–Whitney test, *p* = 0.6991). In neurons excited by LH stimulation, antagonizing DNQX-sensitive AMPA receptors did not unmask GABAergic currents; in neurons inhibited by LH stimulation, antagonizing GABA_A_Rs did not unmask glutamatergic currents. These two observations suggest that glutamatergic and GABAergic LH axons synapsed largely, if not entirely, on distinct groups of pnVTA dopaminergic neuron. There were no obvious differences in the spatial distribution of these two groups and their spontaneous spiking rates were similar (Extended Data Fig. 2-1*C*).

In lateral VTA dopaminergic neurons, LH GABAergic neurons are thought to drive positive reinforcement and place preference through a disynaptic circuit that involves a VTA GABAergic interneuron (Nieh et al., 2016). To determine whether the short latency GABAergic responses evoked in pnVTA dopaminergic neurons by LH stimulation also relied upon local interneurons, two experiments were performed. First, if LH glutamatergic neurons were driving VTA GABAergic interneurons, then LH-evoked GABA_A_R-mediated currents in pnVTA neurons should be lost following the application of ionotropic glutamate receptor antagonists (Fig. 2*G*). However, bath application of DNQX (10** **µM) did not affect the LH-evoked pause in pnVTA dopaminergic neuron spiking (control: 7 ± 7%baseline; +DNQX: 9 ± 9%baseline; *n* = 5 cells; RM one-way ANOVA with Tukey's multiple comparisons, *p* = 0.6152); in contrast, the GABA_A_R antagonist SR (10** **µM) completely blocked the pause (Fig. 2*G*; SR: 105 ± 5%baseline; *p* = 0.0003). To provide an additional test, the effects of optogenetic stimulation of LH axons on pnVTA dopaminergic neurons were assessed in the presence of DNQX, as well as in the presence of tetrodotoxin (TTX, 1** **µM) and 4-aminopyridine (4-AP, 10** **µM) to disrupt conducted activity. In whole-cell recordings, optogenetic activation of LH axons evoked robust IPSCs in this configuration that were sensitive to SR (10** **µM; Fig. 2*H*). Taken together, these results demonstrate that GABAergic and glutamatergic LH neurons innervate distinct subsets of pnVTA dopaminergic neurons.

### MOR signaling inhibited both glutamatergic and GABAergic LH synapses on pnVTA dopaminergic neurons

**Figure 3.**
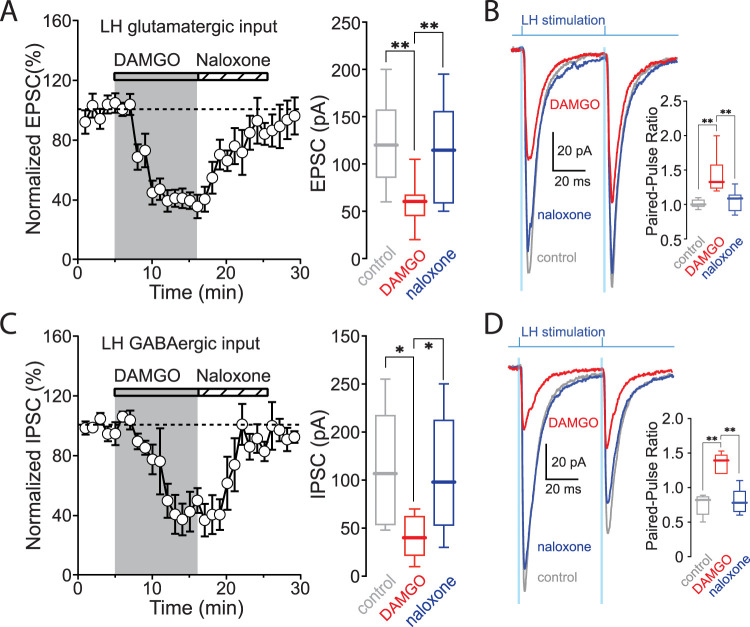
DAMGO inhibited both EPSCs and IPSCs induced by LH stimulation. ***A***, Averaged time course of EPSCs in response to DAMGO. EPSCs were evoked in pnVTA dopaminergic neurons in response to 0.05** **Hz optical stimulation of LH axons. DAMGO (1** **µM) reduced the EPSC amplitude. Naloxone (10** **µM) reversed the inhibition. Data summary (*n* = 9 cells). ***B***, Averaged trace of EPSCs evoked by paired stimuli before (gray) and during (red) DAMGO application and after naloxone application (blue). DAMGO produced paired-pulse facilitation that was blocked by naloxone (*n* = 9 cells). ***C***, Averaged time course of IPSCs in response to DAMGO. DAMGO (1** **µM) reduced the IPSC amplitude. Naloxone (10** **µM) reversed the inhibition. Data summary (*n* = 6 cells). ***D***, Averaged trace of IPSCs evoked by paired stimuli before (gray) and during (red) DAMGO application and after naloxone application (blue). DAMGO produced paired-pulse facilitation that was blocked by naloxone (*n* = 5 cells). The effect of DAMGO in female mice is shown in the Extended Data Figure 3-1.

Optogenetic and pharmacological approaches were used to determine whether LH glutamatergic synapses on pnVTA dopaminergic neurons were modulated by MORs. In ex vivo brain slices, LH axons were optogenetically stimulated while monitoring evoked EPSCs in pnVTA dopaminergic neurons. In the presence of the GABA_A_R antagonist SR, bath application of the MOR agonist d-Ala-*N*-Me-Phe^-^Gly-ol-enkephalin (DAMGO, 1** **µM) significantly suppressed EPSCs (Fig. 3*A*; control: 124 ± 15** **pA; +DAMGO: 59 ± 8** **pA; *n* = 9 cells; RM one-way ANOVA with Tukey's multiple comparisons, *p* = 0.0005). The effects of DAMGO were largely abolished by the MOR antagonist, naloxone (10** **µM; +naloxone: 110 ± 18** **pA; *p* = 0.0072). In addition, the paired-pulse ratio (PPR) of the LH-evoked EPSCs rose after MOR activation (control: 1.02 ± 0.02; +DAMGO: 1.44 ± 0.09; *n* = 9 cells; RM one-way ANOVA with Tukey's multiple comparisons, *p* = 0.0073) and returned to baseline after MOR blockade by naloxone (Fig. 3*B*; +naloxone: 1.06 ± 0.05; *p* = 0.0044).

10.1523/JNEUROSCI.1194-24.2025.f3-1 Figure 3-1 DAMGO inhibited both EPSCs and IPSCs induced by LH stimulation in the female mice. A, Averaged time course of EPSCs in response to DAMGO. EPSCs were evoked in pnVTA dopaminergic neurons in response to 0.05  Hz optical stimulation of LH axons. DAMGO (1  µM) reduced the EPSC amplitude. Naloxone (10  µM) reversed the inhibition. Data summary (n = 5 cells). B, Averaged trace of EPSCs evoked by paired stimuli before (grey) and during (red) DAMGO application and after naloxone application (blue). DAMGO produced paired-pulse facilitation that was blocked by naloxone (n = 5 cells). C, Averaged time course of IPSCs in response to DAMGO. DAMGO (1  µM) reduced the IPSC amplitude. Naloxone (10  µM) reversed the inhibition. Data summary (n = 6 cells). D, Averaged trace of IPSCs evoked by paired stimuli before (grey) and during (red) DAMGO application and after naloxone application (blue). DAMGO produced paired-pulse facilitation that was blocked by naloxone (n = 6 cells). Download Figure 3-1, TIF file.

To assess whether MOR signaling modulated LH GABAergic synapses on pnVTA dopaminergic neurons, IPSCs were recorded as above in the presence of DNQX. LH-evoked IPSCs were suppressed by DAMGO (control: 113 ± 24** **pA; +DAMGO: 41 ± 9** **pA; *n* = 6 cells; RM one-way ANOVA with Tukey's multiple comparisons, *p* = 0.0213). The effects of DAMGO were antagonized by naloxone (Fig. 3*C*; +naloxone: 106 ± 25** **pA; *p* = 0.0343). As with the glutamatergic EPSCs, the PPR of the LH-evoked IPSCs rose after MOR activation (control: 0.76 ± 0.07; +DAMGO: 1.35 ± 0.07; *n* = 5 cells; RM one-way ANOVA with Tukey's multiple comparisons, *p* = 0.0046) and returned to baseline after termination of MOR signaling (Fig. 3*D*; +naloxone: 0.80 ± 0.08; *p* = 0.0050). Thus, both LH glutamatergic and GABAergic synapses on pnVTA dopaminergic neurons were inhibited by MOR signaling.

Although sex differences in opioid addiction and withdrawal have been reported in both animals and humans (Goetz et al., 2021; Dean et al., 2024), no significant differences in the ability of DAMGO to inhibit LH-evoked EPSCs or IPSCs in pnVTA dopaminergic neurons was observed in ex vivo brain slices from female mice (Extended Data Fig. 3-1*A*,*C*; control-EPSC: 147 ± 25** **pA; +DAMGO: 51 ± 13** **pA; *n* = 5 cells; RM one-way ANOVA with Tukey's multiple comparisons, *p* = 0.0103; control-IPSC: 135 ± 39** **pA; +DAMGO: 59 ± 22** **pA; *n* = 6 cells; *p* = 0.0473). As in slices from male mice, naloxone disrupted the modulation of synaptic transmission by MOR activation in slices from female mice (+naloxone, EPSC: 128 ± 22** **pA; *n* = 5 cells; RM one-way ANOVA with Tukey's multiple comparisons, *p* = 0.0109; IPSC: 138 ± 38** **pA; *n* = 6 cells; *p* = 0.0190). Furthermore, MOR activation increased the PPR of EPSCs and IPSCs in slices from female mice (Extended Data Fig. 3-1*B*,*D*; PPR of EPSCs: 1.01 ± 0.11; +DAMGO: 1.40 ± 0.15; *n* = 5 cells; RM one-way ANOVA with Tukey's multiple comparisons, *p* = 0.0216; PPR of IPSCs: 1.12 ± 0.07; +DAMGO: 1.73 ± 0.13; *n* = 6 cells; *p* = 0.0073); naloxone reversed this modulation, as described above (+naloxone, PPR of EPSCs: 1.09 ± 0.12; *n* = 5 cells; RM one-way ANOVA with Tukey's multiple comparisons, *p* = 0.0297; PPR of IPSCs: 1.08 ± 0.08; *n* = 6 cells; *p* = 0.0076). These results show that the MOR modulation of LH terminals in the pnVTA was similar in male and female mice.

### pnVTA dopaminergic neurons also were innervated by vlPAG glutamatergic neurons

Another brain region known to receive aversive input is the vlPAG. As noted above, retrograde viral tracing revealed that neurons in both the vlPAG and adjacent dorsal raphe innervated the pnVTA (Fig. 1*C*).

**Figure 4.**
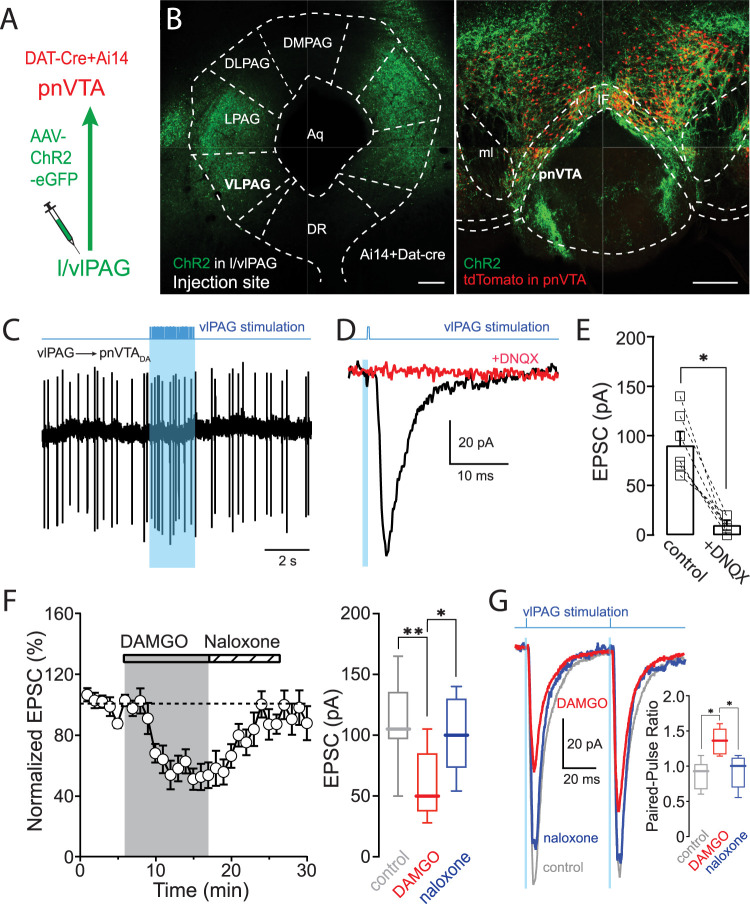
Optogenetic activation of vlPAG axons evoked EPSCs and increased spiking in pnVTA dopaminergic neurons. ***A***, Diagram of viral injection and patch recording. ***B***, After the injection of AAV9-ChR2-EGFP into the vlPAG of the Ai14 × DAT-cre mice, EGFP signal was detected in the vlPAG (injection site, left), and tdTomato and EGFP labeling was detected in the pnVTA (right). Although EYFP expression was observed in the lPAG, the majority of inputs originated from the vlPAG (Fig. 1). Scale bar, 200** **µm. ***C***, Example of optically modulated spiking in pnVTA dopaminergic neurons (*n* = 83 cells). 60% of pnVTA dopaminergic neurons responded to vlPAG stimulation (54%, excited; 6%, inhibited). ***D***, Example of EPSCs (black) evoked in response to 0.05** **Hz optical stimulation of LH axons. DNQX (10** **µM) blocked the EPSCs. ***E***, Data summary (*n* = 7 cells). ***F***, Averaged time course of EPSCs in response to DAMGO. EPSCs evoked in pnVTA dopaminergic neurons in response to 0.05** **Hz optical stimulation of vlPAG axons. DAMGO (1** **µM) reduced the EPSC amplitude. Naloxone (10** **µM) reversed the inhibition. Data summary (*n* = 9 cells, right). ***G***, Averaged trace of EPSCs evoked by paired stimuli before (gray) and during (red) DAMGO application and after naloxone application (blue). DAMGO produced paired-pulse facilitation that was blocked by naloxone (*n* = 6 cells). ***C***, Example of evoked spiking in response to 2** **s optical stimulation (20** **Hz) of vlPAG axons (left). DAMGO (1** **µM; red) reduced the spiking rate, which was reversed by naloxone (10** **µM; blue, right). ***D***, Data summary (*n* = 6 cells). The effect of DAMGO in female mice is shown in the Extended Data Figure 4-1.

To confirm the functional connectivity of vlPAG neurons with pnVTA dopaminergic neurons, a combination of optogenetic and electrophysiological approaches was used (Fig. 4*A*,*B*). Approximately half of the pnVTA dopaminergic neurons (45/83) were excited by optogenetic stimulation of vlPAG axons, whereas only a small fraction (5/83) was inhibited (Fig. 4*C*). Optogenetic burst stimulation of vlPAG axons increased the spiking rate of responsive pnVTA dopaminergic neurons by 438 ± 149% (*n* = 26 cells). To further characterize the vlPAG synapses, whole-cell voltage-clamp recordings were made from pnVTA dopaminergic neurons and vlPAG-evoked EPSCs recorded at a holding potential of −70** **mV. These EPSCs were blocked by DNQX (Fig. 4*D*,*E*; control 91 ± 11** **pA; +DNQX: 10 ± 4** **pA; *n* = 7 cells; Wilcoxon matched-pairs test, *p* = 0.0156). After recording, brain slices were fixed, and confocal imaging was performed to visualize EYFP + vlPAG axons and postsynaptic pnVTA dopaminergic neurons. vlPAG axons were found throughout both the pnVTA and medial PBP (Fig. 4*B*).

10.1523/JNEUROSCI.1194-24.2025.f4-1 Figure 4-1 DAMGO inhibited the EPSCs induced by vlPAG stimulation in the female mice. A, Averaged time course of EPSCs in response to DAMGO. EPSCs evoked in pnVTA dopaminergic neurons in response to 0.05  Hz optical stimulation of vlPAG axons. DAMGO (1  µM) reduced the EPSC amplitude. Naloxone (10  µM) reversed the inhibition. Data summary (n = 5 cells, right). B, Averaged trace of EPSCs evoked by paired stimuli before (grey) and during (red) DAMGO application and after naloxone application (blue). DAMGO produced paired-pulse facilitation that was blocked by naloxone (n = 5 cells). Download Figure 4-1, TIF file.

To determine if MOR signaling modulated vlPAG synapses, the effects of DAMGO on evoked EPSCs were examined. In the presence of the GABA_A_R antagonist SR, DAMGO suppressed vlPAG evoked EPSCs (Fig. 4*F*; control 112 ± 11** **pA; +DAMGO: 61 ± 9** **pA; *n* = 9 cells; RM one-way ANOVA with Tukey's multiple comparisons, *p* = 0.0006). The effects of DAMGO were antagonized by naloxone (10** **µM; +naloxone: 102 ± 10** **pA; *p* = 0.0024). Consistent with a presynaptic locus, the paired-pulse ratio (PPR) of the vlPAG-evoked EPSCs significantly increased after MOR activation (control: 0.88 ± 0.08; +DAMGO: 1.36 ± 0.07; *n* = 6 cells; RM one-way ANOVA with Tukey's multiple comparisons, *p* = 0.0103) and returned to baseline after MOR antagonism with naloxone (Fig. 4*G*; +naloxone: 0.93 ± 0.09; *p* = 0.0209).

In ex vivo brain slices from female mice, DAMGO exerted a similar modulation of vlPAG-evoked EPSCs (Extended Data Fig. 4-1*A*; control: 159 ± 48** **pA; +DAMGO: 63 ± 24** **pA; *n* = 5 cells; RM one-way ANOVA with Tukey's multiple comparisons, *p* = 0.0428). As in slices from male mice, the MOR modulation was blocked by naloxone (+naloxone: 141 ± 37** **pA; *n* = 5 cells; RM one-way ANOVA with Tukey's multiple comparisons, *p* = 0.0173). Furthermore, as described above, the PPR of EPSCs evoked in slices from female mice increased after MOR activation (Extended Data Fig 4-1*B*; control: 0.96 ± 0.07; +DAMGO: 1.26 ± 0.04; *n* = 5 cells; RM one-way ANOVA with Tukey's multiple comparisons, *p* = 0.0170) and returned to baseline after naloxone application (+naloxone, 0.95 ± 0.04; RM one-way ANOVA with Tukey's multiple comparisons, *p* = 0.0066).

### LH and vlPAG glutamatergic inputs converge on the same group of pnVTA dopaminergic neurons

**Figure 5.**
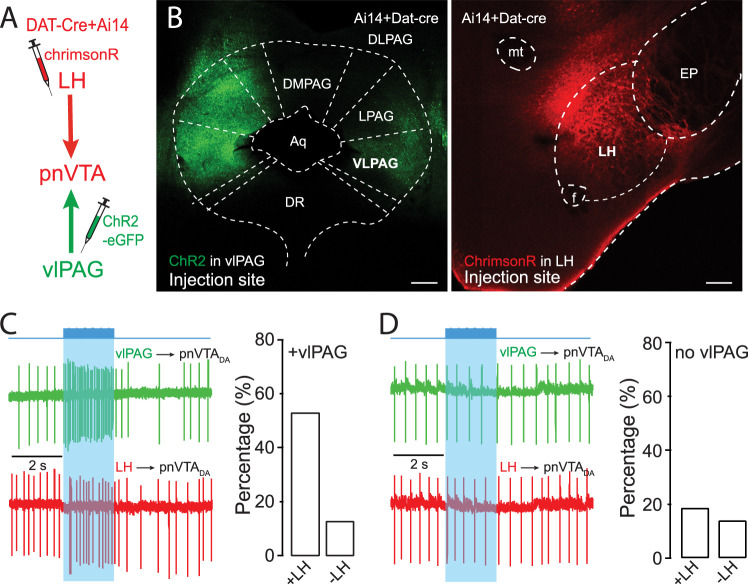
LH and vlPAG glutamatergic terminals converge on the same population of pnVTA dopaminergic neurons. ***A***, Diagram of two-color optogenetic strategy. ***B***, After the injection of AAV9-ChR2-EGFP into the vlPAG and AAV9-chrimsonR into the LH of the Ai14 × DAT-cre mice, EGFP signal was detected in the vlPAG, and chrimsonR signal was detected in the LH. Scale bar, 200** **µm. ***C***, Example of optically evoked spiking in pnVTA dopaminergic neurons (*n* = 56 cells). In pnVTA dopaminergic neurons excited by vlPAG stimulation (*n* = 30 cells), 53% (*n* = 16 cells) of neurons also were excited by LH, ∼ 4 times as many neurons as were inhibited by LH (13%, *n* = 4 cells). ***D***, In pnVTA dopaminergic neurons that did not respond to vlPAG stimulation (*n* = 21 cells), 67% of neurons (*n* = 14 cells) were unresponsive to LH stimulation, 19% (*n* = 4 cells) were excited, and 14% (*n* = 3 cells) were inhibited by LH. The time course of the two-color optogenetics is shown in the Extended Data Figure 5-1.

To determine whether LH and vlPAG glutamatergic axons converged onto the same pnVTA dopaminergic neurons, an optogenetic approach was used. In DAT-Cre X Ai14 mice, the vlPAG was injected with an AAV carrying a ChR2 expression construct, and the LH was injected with an AAV carrying a ChrimsonR expression construct. Four weeks later, brain slices were prepared, and cell-attached recordings were performed from tdTomato-expressing dopaminergic neurons in the pnVTA (Fig. 5*A*). As expected, ChR2 and ChrimsonR were expressed in vlPAG and LH, respectively (Fig. 5*B*). To minimize the coactivation of photoreceptors by light stimulation, ChR2 and ChrimsonR were activated by 430 and 630** **nm light pulses, respectively, at near-threshold optical intensities (5% above threshold; B. Yang et al., 2021). More than half of the neurons sampled (30/56) were excited by optogenetic stimulation of vlPAG axons, whereas less than a tenth (5/56) were inhibited—consistent with the results presented above. Of the pnVTA dopaminergic neurons excited by vlPAG stimulation (*n* = 30 cells), half (16/30) also were excited by optogenetic stimulation of LH axons (Fig. 5*C*, Extended Data Fig. 5-1*A*). Only a small fraction of the neurons excited by vlPAG stimulation were inhibited by LH stimulation (4/30; Extended Data Fig. 5-1*B*), suggesting that LH and vlPAG glutamatergic axons converged on the same set of pnVTA dopaminergic neurons. In contrast, that subset of pnVTA dopaminergic neurons inhibited by vlPAG stimulation (*n* = 5 cells) were invariably inhibited by LH stimulation. In that subset of pnVTA dopaminergic neurons that were not responsive to vlPAG stimulation (*n* = 21 cells), 57% (12/21) were also unresponsive to LH stimulation (Fig. 5*D*). The remaining pnVTA dopaminergic neurons were roughly equally split between neurons that were excited by LH stimulation (4/21) and those that were inhibited (5/21).

10.1523/JNEUROSCI.1194-24.2025.f5-1 Figure 5-1 Convergence of LH and vlPAG inputs in the same dopaminergic neuron. A, The majority of vlPAG-excited dopaminergic neurons were also excited by LH optical stimulation (16 out of 30 responsive cells). Each data point represents the spiking rate normalized to the basal spiking rate (average value of the first 5 data points). The green represents a 430  nm light stimulation (2  s at 20  Hz; 1  ms duration) to activate ChR2 expressed in vlPAG terminals. The red represents a 630  nm light stimulation (2  s at 20  Hz; 1  ms duration) to activate ChrimsonR expressed in LH terminals. The two opsins were stimulated sequentially with a 1-minute interval. B, A small group of vlPAG-excited dopaminergic neurons were inhibited by LH optical stimulation (4 out of 30 responsive cells). Download Figure 5-1, TIF file.

Parsing the data from the LH perspective, 80% of LH-excited neurons were also excited by vlPAG stimulation (16/20). Consistent with the segregation of inputs, roughly 70% of pnVTA dopaminergic neurons that were inhibited by LH stimulation were also inhibited or didn't respond to vlPAG stimulation (10/14).

### pnVTA dopaminergic neurons were innervated by LHb glutamatergic neurons

**Figure 6.**
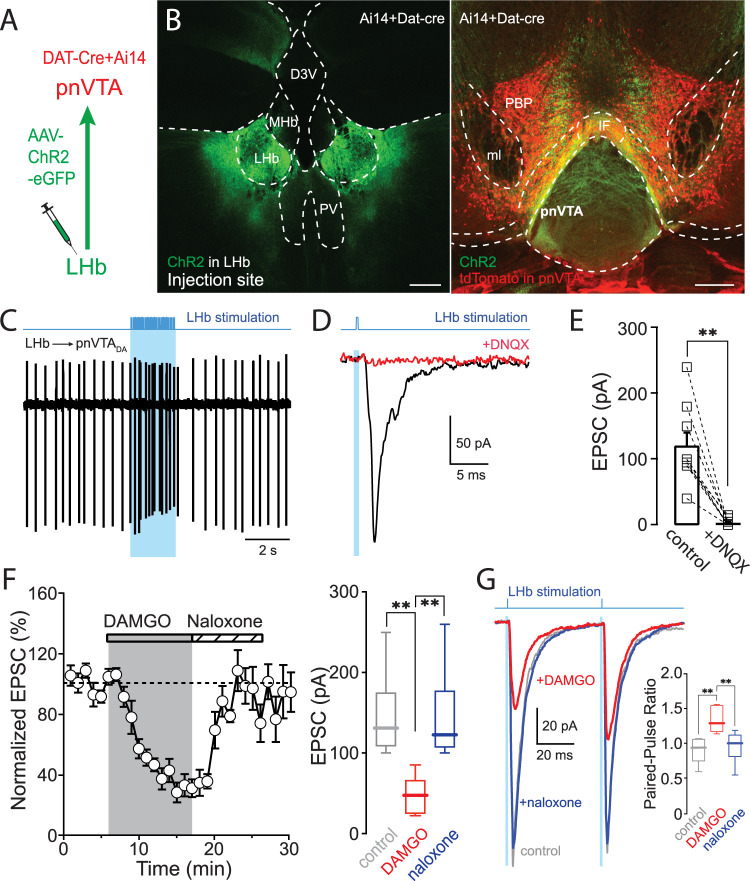
Optogenetic activation of LHb axons evoked EPSCs in pnVTA dopaminergic neurons. ***A***, Diagram of viral injection and patch recording. ***B***, After the injection of AAV9-ChR2-EGFP into the LHb of the Ai14 × DAT-cre mice, EGFP signal was detected in the LHb (injection site, left), and tdTomato and EGFP labeling was detected in the pnVTA (right). Scale bar, 200** **µm. ***C***, Example of optically modulated spiking in pnVTA dopaminergic neurons (*n* = 32 cells; black). 62.5% (*n* = 20 cells) of pnVTA dopaminergic neurons responded to LHb stimulation. ***D***, Example of EPSCs (black) evoked in response to 0.05** **Hz optical stimulation of LH axons. DNQX (10** **µM) blocked the EPSCs (red). ***E***, Data summary (EPSC, *n* = 9 cells). ***F***, Averaged time course of EPSCs in response to DAMGO. EPSCs evoked in pnVTA dopaminergic neurons in response to 0.05** **Hz optical stimulation of LHb axons. DAMGO (1** **µM) reduced the EPSC amplitude. Naloxone (10** **µM) reversed the inhibition. Data summary (*n* = 6 cells, right). ***G***, Averaged trace of EPSCs evoked by paired stimuli before (gray) and during (red) DAMGO application and after naloxone application (blue). DAMGO produced paired-pulse facilitation that was blocked by naloxone (*n* = 5 cells). The effect of DAMGO in female mice is shown in the Extended Data Figure 6-1.

Another potential source of aversive signaling to the pnVTA comes from the LHb (Lammel et al., 2012). As noted above, retrograde viral tracing suggested that neurons in the LHb and the medial habenula (MHb) innervated the pnVTA (Fig. 1*D*); however, the MHb labeling could be a consequence of spread of retroAAV to the interpeduncular nucleus (Montardy et al., 2019). To assess the functional connectivity of these neurons with pnVTA dopaminergic neurons, a combination of optogenetic and electrophysiological approaches was used (Fig. 6*A*). Stereotaxic injection of an AAV carrying a ChR2 expression construct into LHb led to robust axonal labeling throughout the pnVTA but not the lateral PBP (Fig. 6*B*). In ex vivo brain slices, optogenetic stimulation of LHb axons excited ∼60% of the pnVTA dopaminergic neurons sampled (20/32; Fig. 6*C*). Optical burst stimulation of LHb axons increased the spiking rate of responsive pnVTA dopaminergic neurons by 634 ± 140% (*n* = 20 cells). In whole-cell voltage-clamp recordings from pnVTA dopaminergic neurons, optogenetically evoked EPSCs were blocked by DNQX (Fig. 6*D*,*E*; control: 121 ± 20** **pA; +DNQX: 3 ± 2** **pA; *n* = 9 cells; Wilcoxon matched-pairs test, *p* = 0.0039), consistent with a glutamatergic phenotype.

10.1523/JNEUROSCI.1194-24.2025.f6-1 Figure 6-1 DAMGO inhibited the EPSCs induced by LHb stimulation in the female mice. A, Averaged time course of EPSCs in response to DAMGO. EPSCs evoked in pnVTA dopaminergic neurons in response to 0.05  Hz optical stimulation of LHb axons. DAMGO (1  µM) reduced the EPSC amplitude. Naloxone (10  µM) reversed the inhibition. Data summary (n = 6 cells, right). B, Averaged trace of EPSCs evoked by paired stimuli before (grey) and during (red) DAMGO application and after naloxone application (blue). DAMGO produced paired-pulse facilitation that was blocked by naloxone (n = 5 cells). Download Figure 6-1, TIF file.

To determine if MOR signaling modulated LHb synapses, the effects of bath application of DAMGO on evoked EPSCs were examined. In the presence of the GABA_A_R antagonist SR, DAMGO suppressed optogenetically evoked EPSCs (Fig. 6*F*; control: 146 ± 21** **pA; +DAMGO: 48 ± 10** **pA; *n* = 6 cells; RM one-way ANOVA with Tukey's multiple comparisons, *p* = 0.0061). Naloxone antagonized the inhibition produced by DAMGO (+naloxone: 144 ± 24** **pA; *p* = 0.0087). Consistent with a presynaptic locus, the PPR of the LHb-evoked EPSCs increased after MOR activation (control: 0.89 ± 0.09; +DAMGO: 1.34 ± 0.09; *n* = 5 cells; RM one-way ANOVA with Tukey's multiple comparisons, *p* = 0.0012) and returned to baseline after MOR antagonism (Fig. 6*G*; naloxone: 9.94 ± 0.14; *p* = 0.0061).

In ex vivo brain slices from female mice, DAMGO inhibited LHb-induced EPSCs (Extended Data Fig. 6-1*A*; control: 163 ± 34** **pA; +DAMGO: 69 ± 8** **pA; *n* = 6 cells; RM one-way ANOVA with Tukey's multiple comparisons, *p* = 0.0424) and increased the PPR (Extended Data Fig. 6-1*B*; control: 0.94 ± 0.11; +DAMGO: 1.31 ± 0.19; *n* = 5 cells; RM one-way ANOVA with Tukey's multiple comparisons, *p* = 0.0402). As in slices from male mice, naloxone blocked the DAMGO modulation (+naloxone, EPSC: 162 ± 34; *n* = 6 cells; RM one-way ANOVA with Tukey's multiple comparisons, *p* = 0.0434; PPR: 0.94 ± 0.11; *n* = 5 cells; *p* = 0.0401).

### LH, vlPAG, and LHb transmit distinctive types of aversive information to pnVTA

To assess the type of information LH, vlPAG, and LHb transmitted to the pnVTA, an intersectional strategy was used: pnVTA-projecting neurons in each region were retrogradely labeled and then the ability of three different types of aversive stimuli to increase cFos expression in these labeled neurons determined. The three aversive stimuli were tail pinch, predator odor, or an unpleasant odor (formaldehyde).

**Figure 7.**
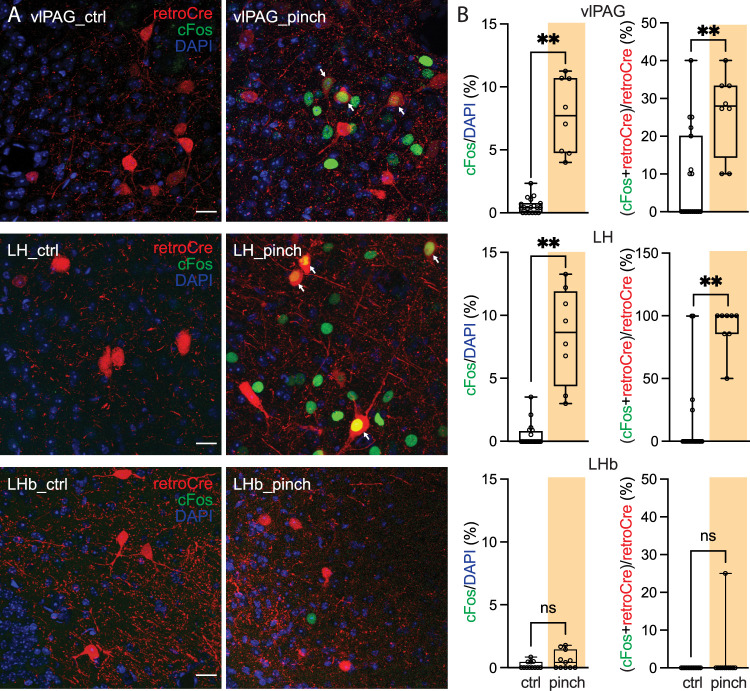
LH and vlPAG neurons projecting to pnVTA encode nociceptive information. The Ai14 mice (*n* = 6) were injected with retrograde AAVrg-Cre into the pnVTA. Mice were allowed for a 30 min acclimatization in the procedure cage. The pinch group (*n* = 3 mice) was given 10 tail pinches (1** **s duration, 60** **s interval) and the control group (*n* = 3) was allowed to move freely for 10 min. Mice were killed for immunohistochemistry 45 min after the tail pinch. Neurons projecting to pnVTA were labeled with tdTomato (red), and the green and the blue were cFos signal and DAPI. ***A***, cFos signal (cFos/DAPI) and pinch-activated neurons projecting to pnVTA [(cFos + retroCre)/retroCre; arrowhead] increased in vlPAG (first row) and LH (second row) after tail pinch. In contrast, no change of cFos was observed in LHb (third row) after pinch stimulation. Scale bar, 20** **µm. ***B***, Data summary (vlPAG-ctrl, *n* = 19 imaging sites; vlPAG-pinch, *n* = 8 imaging sites; LH-ctrl, *n* = 20 imaging sites; LH-pinch, *n* = 8 imaging sites; LHb-ctrl, *n* = 12 imaging sites; LHb-pinch, *n* = 11 imaging sites).

After allowing mice to adapt to the testing chamber for 30 min, mice were subjected to a tail pinch (1** **s duration; 10 repetitions with 60** **s intervals). Forty-five minutes later, mice were killed, and their brains fixed. On the following day, 50** **µm brain slices were prepared for immunostaining. Tail pinch significantly enhanced the expression of cFos in both the LH and vlPAG, but not the LHb (cFos/DAPI; LH-ctrl: 0.46 ± 0.21%, *n* = 20 imaging sites; LH-pinch: 8.38 ± 1.35%, *n* = 8 imaging sites; Mann–Whitney test, *p* < 0.0001; vlPAG-ctrl: 0.54 ± 0.14%, *n* = 19 imaging sites; vlPAG-pinch: 7.71 ± 1.05%, *n* = 8 imaging sites; Mann–Whitney test, *p* < 0.0001; LHb-ctrl: 0.18 ± 0.08%, *n* = 12 imaging sites; LHb-pinch: 1.13 ± 0.17%, *n* = 11 imaging sites; Mann–Whitney test, *p* = 0.1829; Fig. 7*A*,*B*). Moreover, tail pinch increased cFos expression in pnVTA-projecting LH and vlPAG neurons, but not those in the LHb [(cFos + retroCre)/retroCre; LH-ctrl: 12.92 ± 6.96%, *n* = 20 imaging sites; LH-pinch: 90.18 ± 6.18%, *n* = 8 imaging sites; Mann–Whitney test, *p* < 0.0001; vlPAG-ctrl: 8.60 ± 2.79%, *n* = 19 imaging sites; vlPAG-pinch: 26.22 ± 3.84%, *n* = 8 imaging sites; Mann–Whitney test, *p* = 0.0014; LHb-ctrl: 0.00 ± 0.00%, *n* = 12 imaging sites; LHb-pinch: 2.27 ± 2.27%, *n* = 11 imaging sites; Mann–Whitney test, *p* = 0.4783; Fig. 7*A*,*B*].

**Figure 8.**
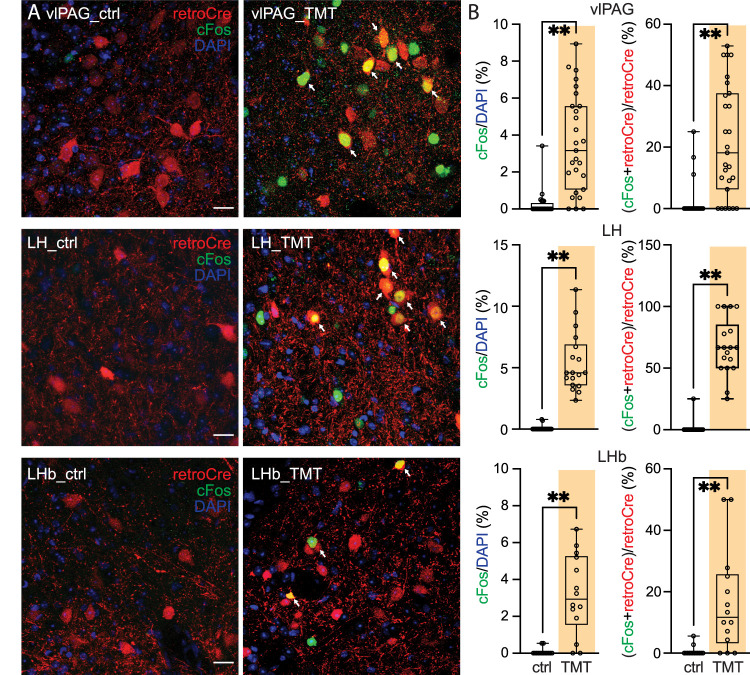
LH, vlPAG, and LHb neurons projecting to pnVTA collaboratively encode predator information. The Ai14 mice (*n* = 6) were injected with retrograde AAVrg-Cre into the pnVTA. Mice were allowed for a 30 min acclimatization in the procedure cage. Mice were exposed to water (control; *n* = 3 mice) or TMT solution (TMT; *n* = 3 mice) for 15 min. Neurons projecting to pnVTA were labeled with tdTomato (red), and the green and the blue were cFos signal and DAPI. ***A***, cFos signal (cFos/DAPI) and TMT-activated neurons projecting to pnVTA [(cFos + retroCre)/retroCre; arrowhead] increased in vlPAG (first row), LH (second row), and LHb (third row) after TMT exposure. Scale bar, 20** **µm. ***B***, Data summary (vlPAG-ctrl, *n* = 24 imaging sites; vlPAG-TMT, *n* = 27 imaging sites; LH-ctrl, *n* = 24 imaging sites; LH-TMT, *n* = 18 imaging sites; LHb-ctrl, *n* = 14 imaging sites; LHb-TMT, *n* = 14 imaging sites).

To complement the tail pinch experiments, mice were exposed to a stressful predator odor, 2,5-dihydro-2,3,5-trimethylthiazoline (TMT). Specifically, mice were exposed to water or TMT solution for 15 min. During exposure, mice moved away from TMT solution and exhibited freezing behavior (Pérez-Gómez et al., 2015). Approximately 45 min later, mice were killed and brains fixed. The next day, brains were processed as described above. TMT exposure increased cFos expression in LH, vlPAG, and LHb (LH-ctrl: 0.06 ± 0.04%, *n* = 24 imaging sites; LH-TMT: 5.39 ± 0.57%, *n* = 18 imaging sites; Mann–Whitney test, *p* < 0.0001; vlPAG-ctrl: 0.25 ± 0.15%, *n* = 24 imaging sites; vlPAG-TMT: 3.58 ± 0.51%, *n* = 27 imaging sites; Mann–Whitney test, *p* < 0.0001; LHb-ctrl: 0.08 ± 0.05%, *n* = 14 imaging sites; LHb-TMT: 3.17 ± 0.58%, *n* = 14 imaging sites; Mann–Whitney test, *p* < 0.0001; Fig. 8*A*). Moreover, TMT increased cFos expression in pnVTA-projecting neurons in all three regions (Fig. 8*A*,*B*; LH-ctrl: 1.04 ± 1.04%, *n* = 24 imaging sites; LH-TMT: 67.08 ± 0.57%, *n* = 18 imaging sites; Mann–Whitney test, *p* < 0.0001; vlPAG-ctrl: 2.20 ± 1.28%, *n* = 24 imaging sites; vlPAG-TMT: 21.91 ± 3.52%, *n* = 27 imaging sites; Mann–Whitney test, *p* < 0.0001; LHb-ctrl: 0.60 ± 0.43%, *n* = 14 imaging sites; LHb-TMT: 16.67 ± 4.45%, *n* = 14 imaging sites; Mann–Whitney test, *p* = 0.0001).

**Figure 9.**
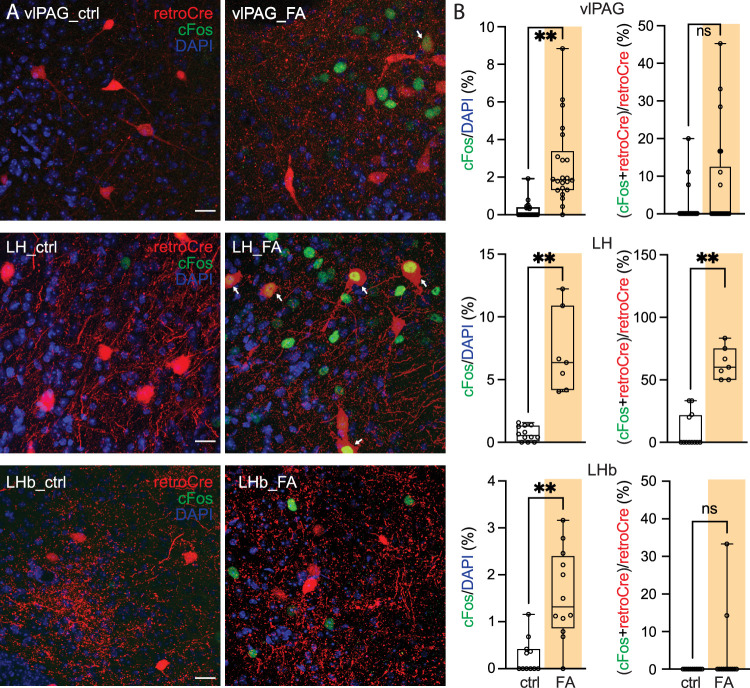
LH neurons projecting to pnVTA respond to an unpleasant odor FA. The Ai14 mice (*n* = 6) were injected with retrograde AAVrg-Cre into the pnVTA. Mice were allowed for a 30 min acclimatization in the procedure cage. Mice were exposed to water (control; *n* = 3 mice) or 6% FA solution (FA; *n* = 3 mice) for 15 min. Neurons projecting to pnVTA were labeled with tdTomato (red), and the green and the blue were cFos signal and DAPI. ***A***, cFos signal (cFos/DAPI) increased in vlPAG (first row), LH (second row), and LHb (third row) after FA exposure. Only in the LH did TMT exposure increase the number of FA-activated neurons projecting to the pnVTA [(cFos + retroCre)/retroCre; arrowhead]. Scale bar, 20** **µm. ***B***, Data summary (vlPAG-ctrl, *n* = 22 imaging sites; vlPAG-FA, *n* = 22 imaging sites; LH-ctrl, *n* = 12 imaging sites; LH-FA, *n* = 7 imaging sites; LHb-ctrl, *n* = 11 imaging sites; LHb-FA, *n* = 12 imaging sites).

To complement the tail pinch and predator odor experiments, mice were exposed to formaldehyde (FA), which was unpleasant but dissimilar to the other stimuli. Specifically, mice were exposed to either water or FA solution for 15 min. During exposure, mice approached the FA solution and then moved away, as reported previously (de Jong et al., 2019). Mice were killed and brains fixed 45 min later. The next day, brains were processed as described above. FA exposure significantly enhanced cFos expression in all three regions (cFos/DAPI; LH-ctrl: 0.69 ± 0.17%, *n* = 12 imaging sites; LH-FA: 7.11 ± 1.21%, *n* = 7 imaging sites; Mann–Whitney test, *p* < 0.0001; vlPAG-ctrl: 0.23 ± 0.09%, *n* = 22 imaging sites; vlPAG-FA: 2.63 ± 0.45%, *n* = 22 imaging sites; Mann–Whitney test, *p* < 0.0001; LHb-ctrl: 0.27 ± 0.11%, *n* = 11 imaging sites; LHb-FA: 1.57 ± 0.27%, *n* = 12 imaging sites; Mann–Whitney test, *p* = 0.0005; Fig. 9*A*,*B*). However, FA significantly increased cFos expression in pnVTA projection neurons only in the LH (LH-ctrl: 9.07 ± 4.02%, *n* = 12 imaging sites; LH-FA: 63.16 ± 4.76%, *n* = 7 imaging sites; Mann–Whitney test, *p* < 0.0001; vlPAG-ctrl: 1.76 ± 1.05%, *n* = 22 imaging sites; vlPAG-FA: 7.25 ± 2.78%, *n* = 22 imaging sites; Mann–Whitney test, *p* = 0.1059; LHb-ctrl: 0.00 ± 0.00%, *n* = 11 imaging site; LHb-FA: 3.97 ± 2.92%, *n* = 12 imaging sites; Mann–Whitney test, *p* = 0.4783). Taken together, these experiments demonstrate that LH, vlPAG, and LHb convey aversive information to pnVTA dopaminergic neurons but that the quality of the information being conveyed depends upon the region.

## Discussion

Three conclusions can be drawn from our studies. First, three brain regions activated by aversive stimuli—the LH, vlPAG, and LHb—innervate pnVTA dopaminergic neurons. Second, glutamatergic neurons in these aversion-related regions innervate a largely overlapping population of pnVTA dopaminergic neurons; in contrast, LH GABAergic projections to pnVTA target a different population of dopaminergic neurons. Third, presynaptic MORs suppress transmission from all three regions to pnVTA dopaminergic neurons. Thus, unlike the circuitry in the lateral VTA where MORs disinhibit dopaminergic neurons by suppressing their GABAergic innervation, in the pnVTA region, MORs directly suppress both glutamatergic and GABAergic synaptic transmission from aversion-driven regions to dopaminergic neurons. Given the linkage of pnVTA dopaminergic neurons to aversion and pain, our studies define a neural substrate in the mesolimbic circuitry for the negative reinforcing value of opioids that is likely to play a significant role in addiction.

### Three brain regions activated by aversive stimuli innervate the pnVTA

Previous connectome mapping studies have shown that LH, vlPAG, and LHb project to VTA (Watabe-Uchida et al., 2012). Our work extends this mapping in two key ways. First, our studies demonstrate that neurons in each of these three projection sites convey information about aversive events to pnVTA. LH neurons projecting to the pnVTA were activated by a broad range of aversive stimuli—tail pinch, predator odor, and FA—whereas the stimuli activating vlPAG and LHb projection neurons were more restricted (vlPAG: pinch, predator odor; LHb: predator odor). Second, using optogenetic approaches, our work demonstrated that LH, vlPAG, and LHb neurons not only project to the pnVTA, but they innervate dopaminergic neurons there. Roughly 40% of the pnVTA dopaminergic neurons were excited by optogenetic stimulation of LH axons, whereas a similar percentage were inhibited. Pharmacological analysis demonstrated these responses were attributable to glutamatergic and GABAergic synapses, respectively. Importantly, these two populations were essentially nonoverlapping, suggesting that nearly all pnVTA dopaminergic neurons were innervated by one or the other type of LH neuron. Although vlPAG glutamatergic neurons innervated a similar percentage of pnVTA dopaminergic neurons (∼50%), GABAergic responses evoked by optogenetic stimulation of vlPAG were rarely observed (∼10%). Dual color optogenetic experiments found that the pnVTA dopaminergic neurons innervated by LH and vlPAG glutamatergic neurons were overlapping. As for LH and vlPAG glutamatergic neurons, optogenetic stimulation of LHb glutamatergic neurons evoked clear responses in about half of the pnVTA dopaminergic neurons. These results suggest that a subset of the pnVTA dopaminergic neurons receive convergent glutamatergic innervation from LH, vlPAG, and LHb, with a distinct subgroup of the population being innervated by LH GABAergic neurons.

This inferred connectivity is consistent with other observations. Dopaminergic pnVTA neurons innervate the dorsomedial shell of the NAc (dmNAc) and the ventromedial shell of the NAc (vmNAc). Neurons in dmNAc are responsive to reward-related events whereas those in the vmNAc are responsive to aversive events (de Jong et al., 2019). Thus, given the ability of aversive events to drive activity in LH, vlPAG, and LHb glutamatergic neurons (Keay et al., 2002; Tovote et al., 2016; Li et al., 2021; Siemian et al., 2021), it seems likely that these neurons innervate pnVTA dopaminergic neurons projecting to the vmNAc, medial prefrontal cortex, or basolateral amygdala (Verharen et al., 2020).

**Figure 10.**
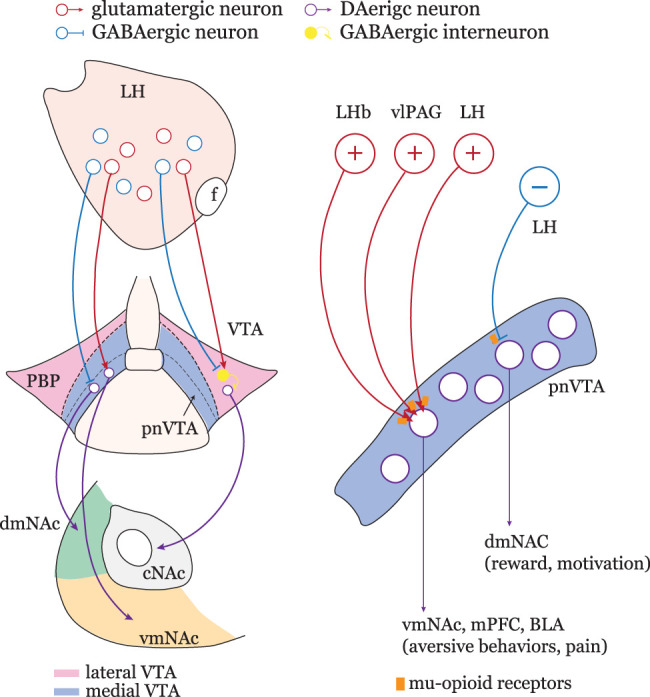
Schematic wiring diagram of the VTA circuitry. Right, LH glutamatergic neurons innervate pnVTA dopaminergic neurons that project to aversion-related regions, including the vmNAc, mPFC, and BLA. LH GABAergic neurons innervate a different subgroup of pnVTA dopaminergic neurons, which project to reward-related dmNAc. In the lateral VTA, both LH glutamatergic and GABAergic neurons target VTA interneurons which inhibit dopaminergic neurons. These dopaminergic neurons project to reward-related cNAc. Left, LHb, vlPAG, and LH glutamatergic neurons preferentially target the same pnVTA dopaminergic population. The activation of MORs suppresses the glutamate release from LHb, vlPAG, and LH terminals and suppresses GABA release from LH as well. mPFC, medial prefrontal cortex; BLA, basolateral amygdala.

The information being conveyed by LH GABAergic neurons innervating the pnVTA is less clear. Previous work suggests that LH GABAergic neurons projecting to the lateral VTA innervate GABAergic interneurons and convey reward-related signals (Nieh et al., 2016). So, activation of these LH GABAergic projection neurons leads to disinhibition of lateral VTA dopaminergic neurons projecting to the cNAc (Ambroggi et al., 2011). However, the LH GABAergic neurons projecting to the pnVTA do not rely upon interneuron inversion of their signaling. Our working hypothesis is that these LH GABAergic neurons are driven by aversive events, but this will require additional study (Fig. 10).

### Presynaptic MORs suppressed synaptic modulation of pnVTA dopaminergic neurons

A key observation in our experiments was that presynaptic MORs suppressed transmitter release from LH, vlPAG, and LHb synapses on pnVTA dopaminergic neurons. The MOR agonist DAMGO significantly reduced the amplitude of LH, vlPAG, and LHb evoked glutamatergic EPSCs. DAMGO also reduced the amplitude of LH-evoked GABAergic IPSCs. Often, the effects of DAMGO on synaptic transmission persisted after its removal. Invariably, the persistent modulation was reversed by naloxone, arguing that they were mediated by persistent MOR signaling, rather than a long-term change in transmitter release. The inhibition of transmitter release was accompanied by an elevation in paired-pulse ratio, consistent with a presynaptic locus of action. The inference that presynaptic MORs suppress transmitter release from these three converging sites is consistent with studies demonstrating that all three regions express MORs (Margolis and Fields, 2016; Zhang et al., 2020).

These results have implications for understanding the addictive potential of opioids. Long-term use of opioids leads to neuronal adaptations in the central nervous system (CNS) and mesencephalic dopaminergic neurons in particular (Kosten and Baxter, 2019). The classical view is that opioids activate MORs expressed by GABAergic neurons that suppress the activity of VTA dopaminergic neurons, leading to an elevation in their excitability and release of dopamine in the NAc (Doyle and Mazei-Robison, 2021). As with other abused drugs, the opioid-induced elevation in NAc dopamine release is widely viewed as reinforcing (Zhou et al., 2022; Luján et al., 2023). With repeated administration of opioids, tolerance develops causing a persistent depression in dopamine release with drug withdrawal, which then drives reinstatement (Fox et al., 2017). Our studies demonstrate that there is a complementary mechanism of opioid action on pnVTA dopaminergic neurons that are excited by aversive stimuli. The existing data is consistent with the conjecture that these neurons project to the vmNAc and limbic cortices—regions linked to aversion (Lammel et al., 2012; de Jong et al., 2019; Ren et al., 2021). MORs act presynaptically to suppress the ability of aversive signaling pathways to activate these pnVTA dopaminergic neurons. Thus, in addition to the positive reinforcement through the disinhibition of reward-related dopaminergic neurons in the lateral VTA, in an aversive state such as that induced by chronic pain, opioids may reduce the excitation of aversion-related pnVTA dopaminergic neurons, thereby alleviating the resulting pain.

As noted above, one unanswered question is the functional role of LH GABAergic neurons innervating pnVTA dopaminergic neurons. This direct synaptic couple was inhibited by MOR signaling, just as was the glutamatergic synapse. On the face of it, the MOR-mediated inhibition of the LH GABAergic suggests that it is conveying aversive information, not reward-related information. If this was the case, then the LH GABAergic projections to the pnVTA and the lateral VTA would be distinct. In support of the proposition that there is a distinct subset of pnVTA dopaminergic neurons coding reward, Corre et al. found a subset of neurons in this region that was activated by heroin (Corre et al., 2018).

### Implications for central mechanisms of pain and opioid addiction

Mesolimbic dopaminergic neurons are clearly implicated in opioid reward and addiction (Pascoli et al., 2015; Juarez and Han, 2016; Monroe and Radke, 2023). Most of this work has focused on the modulation of lateral VTA dopaminergic neurons innervating the cNAc and other limbic regions. Opioids and MOR signaling disinhibits this group of dopaminergic neurons, leading to dopamine release in the cNAc and reward (Ambroggi et al., 2011; Nieh et al., 2016). With repeated opioid administration, tolerance develops, leading to diminished opioid-mediated dopamine release and ultimately dependence (Kosten and Baxter, 2019). When opioids are withdrawn after chronic treatment, lateral VTA dopaminergic neurons are inhibited, leading to dysphoria and craving (Bonci and Williams, 1997; Kenny et al., 2006).

The pnVTA dopaminergic neurons appear to be a node in a parallel circuitry in which opioids and MOR signaling directly suppress aversive signaling. By directly inhibiting synaptic transmission in the pnVTA, MORs should reduce the ability of aversive events to stimulate this subtype of dopaminergic neurons. It is highly likely that this circuitry plays an important role in the ability of opioids to diminish pain sensation and to act as negative reinforcers (Weiss et al., 2014; Koob, 2020). Moreover, given that it is likely that this circuitry develops tolerance with chronic opioid exposure, it might become hyperactive with withdrawal. Indeed, relapse following opioid withdrawal in humans is reportedly pushed largely by the aversive state it creates (Fouyssac et al., 2022; Monroe and Radke, 2023). While this opioid-driven aversive state undoubtedly has a strong somatic component (Kosten and Baxter, 2019; Strang et al., 2020), opioid-induced sensitization of central circuitry is also likely to play a key role. Indeed, recent work by Chaudun et al. (2024) revealed that MOR knockdown in the central nucleus of the amygdala blunted the aversive consequences of fentanyl withdrawal. Our expectation is that knocking down MORs in LH, vlPAG, and LHb neurons projecting to the pnVTA would have similar effects. This recognition could lead to alternative strategies for managing chronic pain, as well as opioid withdrawal.
