# Supplementary figures and images for: Presynaptic Mu Opioid Receptors Suppress the Functional Connectivity of Ventral Tegmental Area Dopaminergic Neurons with Aversion-Related Brain Regions
Source: J Neurosci. 2025 Jun 13;45(28):e1194242025. doi: 10.1523/JNEUROSCI.1194-24.2025 (PMC12244320; doi:10.1523/JNEUROSCI.1194-24.2025)

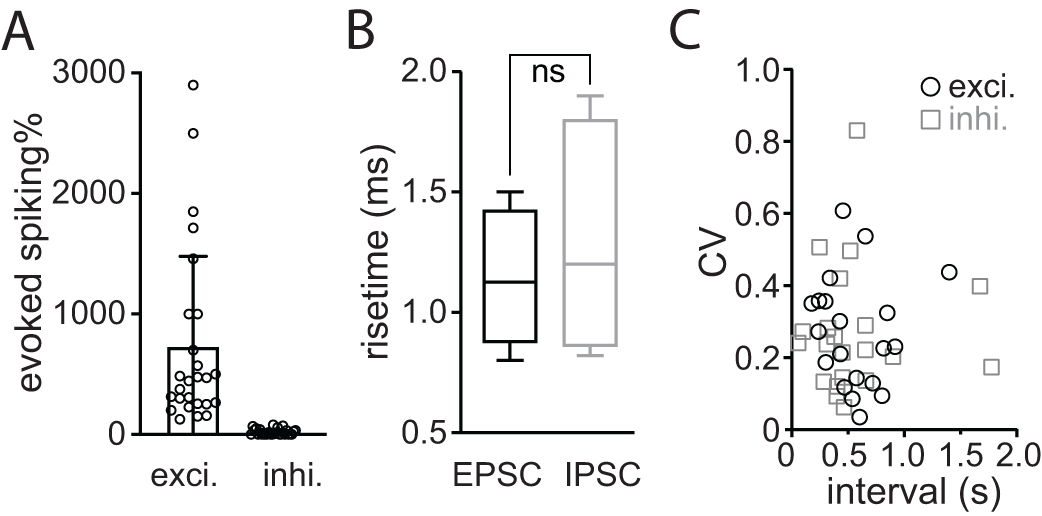

Supplement: Figure 2-1 — The characteristics of LH inputs. A, Quantification of the spiking rate induced by LH stimulation. Excited (n = 26 cells) and inhibited (n = 28 cells) spiking in response to 2 s optical stimulation (40 pulses at 20 Hz; 1 ms duration) of LH axons. Each spiking rate was normalized to the baseline spiking rate. B, The 10-90% rise time of the EPSCs (n = 6 cells) and IPSCs (n = 5 cells). C, Coefficient of variation (CV) - mean plot of the pace-making interval (black, excited, n = 20 cells; gray, inhibited, n = 21 cells). Download Figure 2-1, TIF file. [file jneuro-45-e1194242025-s001.tif]

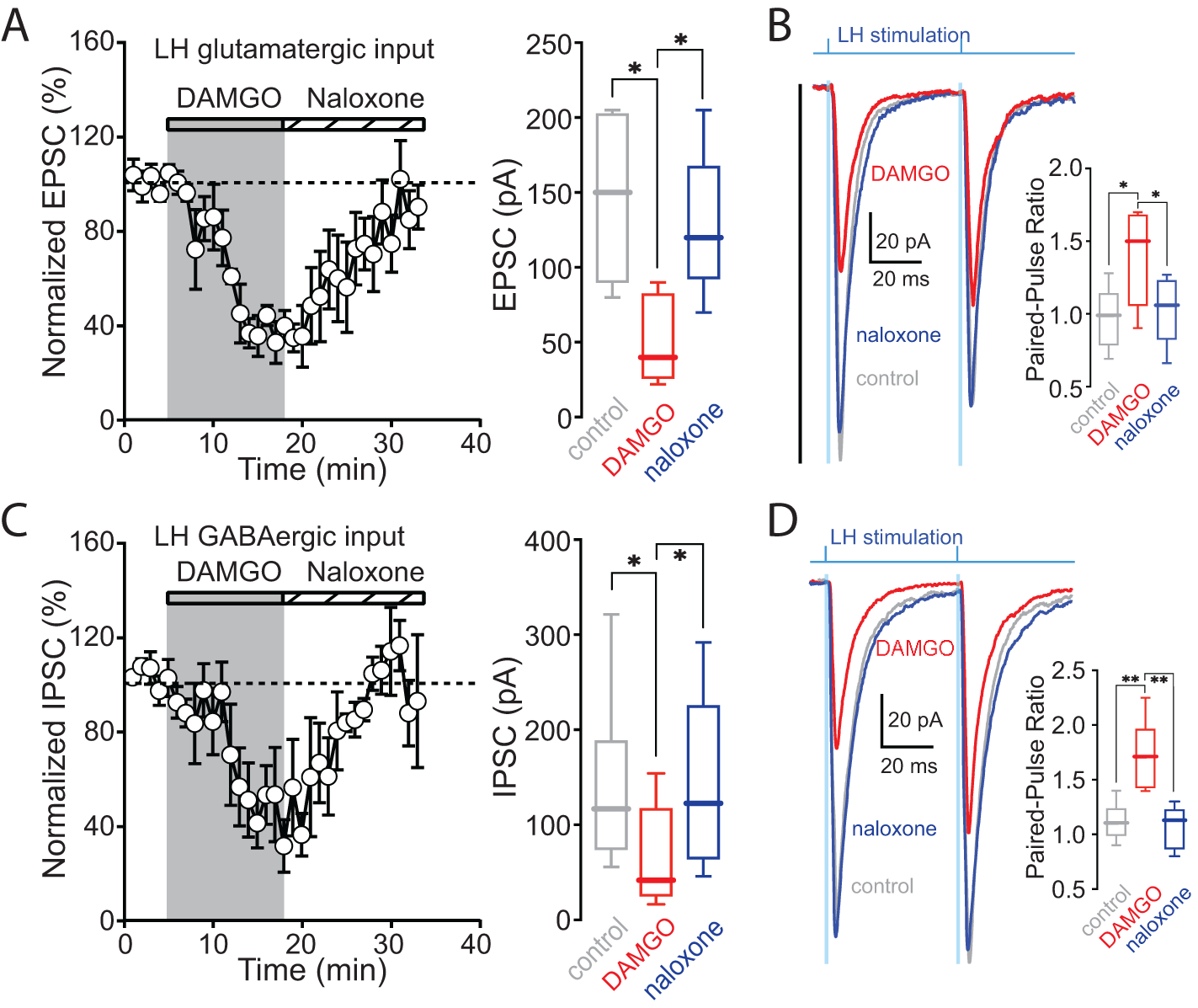

Supplement: Figure 3-1 — DAMGO inhibited both EPSCs and IPSCs induced by LH stimulation in the female mice. A, Averaged time course of EPSCs in response to DAMGO. EPSCs were evoked in pnVTA dopaminergic neurons in response to 0.05 Hz optical stimulation of LH axons. DAMGO (1 µM) reduced the EPSC amplitude. Naloxone (10 µM) reversed the inhibition. Data summary (n = 5 cells). B, Averaged trace of EPSCs evoked by paired stimuli before (grey) and during (red) DAMGO application and after naloxone application (blue). DAMGO produced paired-pulse facilitation that was blocked by naloxone (n = 5 cells). C, Averaged time course of IPSCs in response to DAMGO. DAMGO (1 µM) reduced the IPSC amplitude. Naloxone (10 µM) reversed the inhibition. Data summary (n = 6 cells). D, Averaged trace of IPSCs evoked by paired stimuli before (grey) and during (red) DAMGO application and after naloxone application (blue). DAMGO produced paired-pulse facilitation that was blocked by naloxone (n = 6 cells). Download Figure 3-1, TIF file. [file jneuro-45-e1194242025-s002.tif]

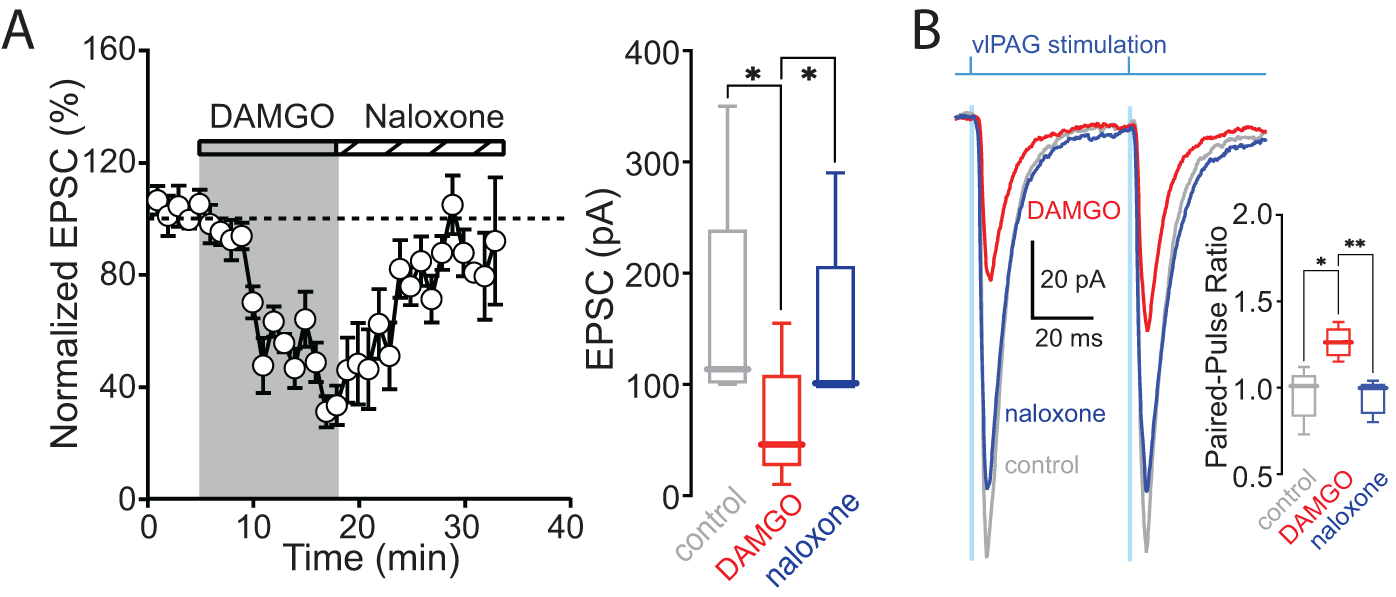

Supplement: Figure 4-1 — DAMGO inhibited the EPSCs induced by vlPAG stimulation in the female mice. A, Averaged time course of EPSCs in response to DAMGO. EPSCs evoked in pnVTA dopaminergic neurons in response to 0.05 Hz optical stimulation of vlPAG axons. DAMGO (1 µM) reduced the EPSC amplitude. Naloxone (10 µM) reversed the inhibition. Data summary (n = 5 cells, right). B, Averaged trace of EPSCs evoked by paired stimuli before (grey) and during (red) DAMGO application and after naloxone application (blue). DAMGO produced paired-pulse facilitation that was blocked by naloxone (n = 5 cells). Download Figure 4-1, TIF file. [file jneuro-45-e1194242025-s003.tif]

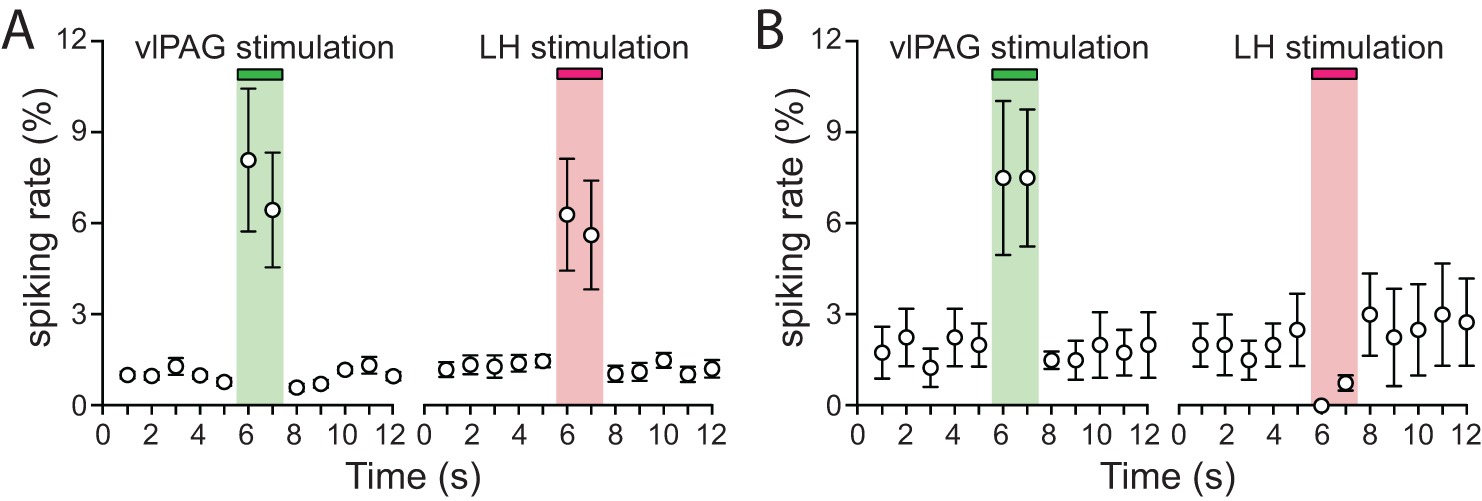

Supplement: Figure 5-1 — Convergence of LH and vlPAG inputs in the same dopaminergic neuron. A, The majority of vlPAG-excited dopaminergic neurons were also excited by LH optical stimulation (16 out of 30 responsive cells). Each data point represents the spiking rate normalized to the basal spiking rate (average value of the first 5 data points). The green represents a 430 nm light stimulation (2 s at 20 Hz; 1 ms duration) to activate ChR2 expressed in vlPAG terminals. The red represents a 630 nm light stimulation (2 s at 20 Hz; 1 ms duration) to activate ChrimsonR expressed in LH terminals. The two opsins were stimulated sequentially with a 1-minute interval. B, A small group of vlPAG-excited dopaminergic neurons were inhibited by LH optical stimulation (4 out of 30 responsive cells). Download Figure 5-1, TIF file. [file jneuro-45-e1194242025-s004.tif]

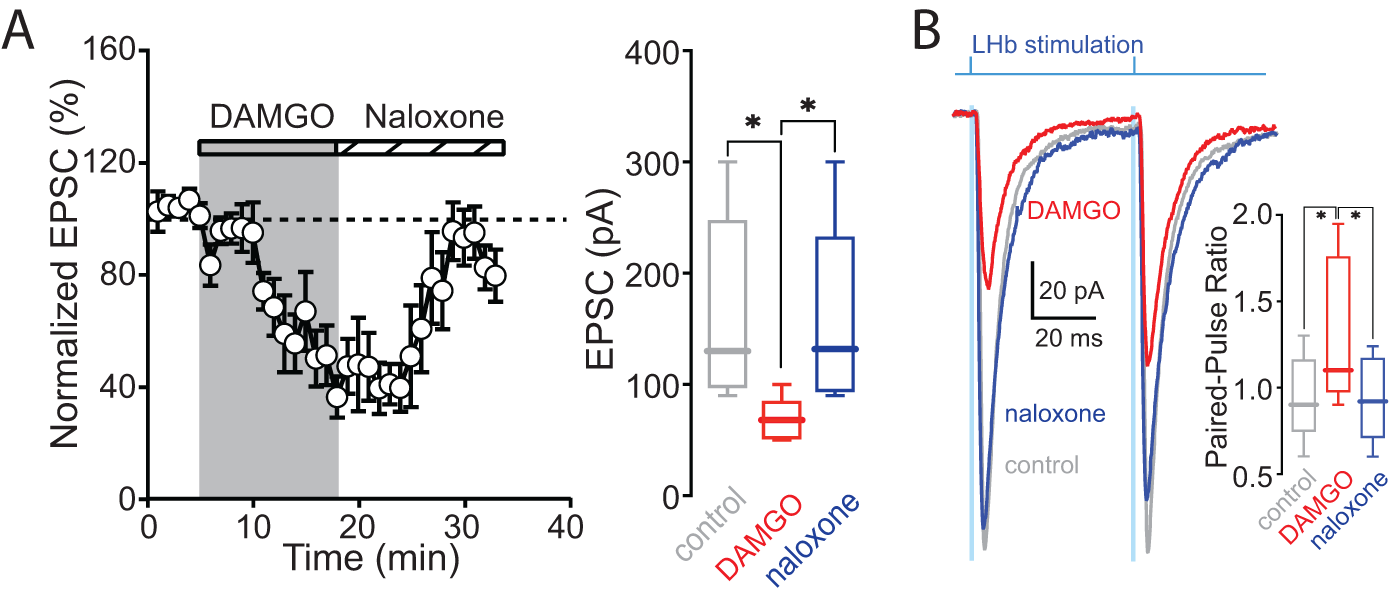

Supplement: Figure 6-1 — DAMGO inhibited the EPSCs induced by LHb stimulation in the female mice. A, Averaged time course of EPSCs in response to DAMGO. EPSCs evoked in pnVTA dopaminergic neurons in response to 0.05 Hz optical stimulation of LHb axons. DAMGO (1 µM) reduced the EPSC amplitude. Naloxone (10 µM) reversed the inhibition. Data summary (n = 6 cells, right). B, Averaged trace of EPSCs evoked by paired stimuli before (grey) and during (red) DAMGO application and after naloxone application (blue). DAMGO produced paired-pulse facilitation that was blocked by naloxone (n = 5 cells). Download Figure 6-1, TIF file. [file jneuro-45-e1194242025-s005.tif]
